# Imaging the microstructure of lithium and sodium metal in anode-free solid-state batteries using electron backscatter diffraction

**DOI:** 10.1038/s41563-024-02006-8

**Published:** 2024-09-23

**Authors:** Till Fuchs, Till Ortmann, Juri Becker, Catherine G. Haslam, Maya Ziegler, Vipin Kumar Singh, Marcus Rohnke, Boris Mogwitz, Klaus Peppler, Linda F. Nazar, Jeff Sakamoto, Jürgen Janek

**Affiliations:** 1 Institute of Physical Chemistry and Center for Materials Research, Justus Liebig University Giessen, Giessen, Germany; 2 Department of Materials Science and Engineering, University of Michigan, Ann Arbor, MI USA; 3 Department of Mechanical Engineering, University of Michigan, Ann Arbor, MI USA; 4 Department of Chemistry and the Waterloo Institute for Nanotechnology, University of Waterloo, Waterloo, Ontario Canada; 5 Materials Department, University of California Santa Barbara, Santa Barbara, CA USA

**Keywords:** Batteries, Characterization and analytical techniques, Batteries

## Abstract

‘Anode-free’ or, more fittingly, metal reservoir-free cells could drastically improve current solid-state battery technology by achieving higher energy density, improving safety and simplifying manufacturing. Various strategies have been reported so far to control the morphology of electrodeposited alkali metal films to be homogeneous and dense, but until now, the microstructure of electrodeposited alkali metal is unknown, and a suitable characterization route is yet to be identified. Here we establish a reproducible protocol for characterizing the size and orientation of metal grains in differently processed lithium and sodium samples by a combination of focused ion beam and electron backscatter diffraction. Electrodeposited films at Cu|Li_6.5_Ta_0.5_La_3_Zr_1.5_O_12_, steel|Li_6_PS_5_Cl and Al|Na_3.4_Zr_2_Si_2.4_P_0.6_O_12_ interfaces were characterized. The analyses show large grain sizes (>100 µm) within these films and a preferential orientation of grain boundaries. Furthermore, metal growth and dissolution were investigated using in situ electron backscatter diffraction, showing a dynamic grain coarsening during electrodeposition and pore formation within grains during dissolution. Our methodology and results deepen the research field for the improvement of solid-state battery performance through a characterization of the alkali metal microstructure.

## Abstract (web summary)

The microstructure of alkali metals in anode-free cells is currently unknown. Here, using a combination of focused ion beam and electron backscatter diffraction, this morphology is investigated, with in situ scanning electron microscopy showing dynamic grain coarsening during electrodeposition.

## Main

Solid-state batteries (SSBs) have gained substantial attention for their potential to surpass lithium-ion batteries as advanced energy storage devices^1–3^. Major advancement is expected by the successful implementation of lithium metal anodes in SSBs, enabled by chemically stable solid electrolytes (SEs)^4,5^, and owing to lithium’s high theoretical specific capacity of 3,861 mAh g^−1^ and low redox potential of −3.04 V. Similar advantages are expected by the use of sodium metal anodes in sodium-based SSBs^6–8^.

‘Anode-free’ or, more fittingly, reservoir-free cells (RFCs) emerged as an alternative to using alkali metal foils for cell fabrication^9–11^. Instead, the metal is deposited onto a specially designed current collector (CC) in the first charging step, eliminating the need for costly handling of reactive alkali metal foils. This approach increases energy density by reducing unnecessary weight while also simplifying fabrication and enhancing safety during storage.

The main challenge of RFCs is controlling the morphology and microstructure of plated alkali metal at the CC|SE interface. Strategies to control the alkali metal layer include specialized plating protocols, engineered CC materials, seed layers and applying pressure during plating^11–17^. Despite intense investigation of the morphology of the metal deposited in RFCs^11–13,18^, its microstructure, including the grain size and orientation, is yet completely unknown^19,20^. Based on previous work, we expect that the microstructure will impact the anode performance by influencing, for example, the pore formation and spatially inhomogeneous plating^19,21,22^.

Another unknown effect concerns alkali metal grain growth during storage at room temperature. Typically, metals show microstructural changes at homologous temperatures (*T*_H_) of around 0.4–0.6 (ref. ^23^). As *T*_H_(Li) = 0.61 and *T*_H_(Na) = 0.80 at room temperature exceed this, it is reasonable to assume that the microstructure of alkali metals is always close to equilibrium, given enough time to anneal. Recent investigations of lithium suggest that its microstructure is actually tunable by a different thermal processing, suggesting slow grain coarsening^21,22^. Interestingly, electrodeposited silver is initially nanocrystalline but anneals during the first hours until grain growth stops at around 40 µm, even at a much lower *T*_H_(Ag) of 0.23 at room temperature^24,25^. It is possible that the initially stated rule of thumb about recrystallization at *T*_H_ > 0.4 may not hold for lithium and sodium.

The most suitable technique to analyse the microstructure of metals and its evolution is electron backscatter diffraction (EBSD). EBSD offers quantitative information on the grain size and orientation, grain boundaries and possibly even dislocations and strain within large single grains^26–28^. However, a very well-defined sample surface with regard to its flatness, crystallinity and chemical composition is required. Thin (<50 nm) degradation layers typically found on lithium^29,30^ mask the electron diffraction pattern due to the very low probe depth of EBSD (~20 nm). Very few EBSD analyses of lithium foils^26,31–33^ exist so far, and to the best of our knowledge, no data have been reported for sodium or electrodeposited alkali metals.

To solve this issue, this work presents a protocol to analyse the microstructure of lithium and sodium foils as well as deposited films in RFCs using EBSD. The success relies on operation in inert gas or high vacuum in all steps, a delicate focused ion beam (FIB) cutting/polishing under cryogenic conditions, and an EBSD system with high sensitivity. First, the results presented here indicate that substantial grain growth of lithium and sodium occurs neither during room temperature storage despite the high *T*_H_, nor during cryogenic FIB preparation. Second, we analyse the microstructure of lithium electrodeposited at Cu|Li_6.5_Ta_0.5_La_3_Zr_1.5_O_12_ (Cu|LLZO) and stainless-steel|Li_6_PS_5_Cl (SS|LPSCl) interfaces as well as sodium plated at a carbon-coated Al|Na_3.4_Zr_2_Si_2.4_P_0.6_O_12_ (Al|NZSP) interface, showing distinctly columnar growth of large grains. These observations offer the first views into the electrochemical growth of unexpected large metal grains and open up a multitude of options for follow-up work on the correlation of electrochemical performance and microstructure of alkali metal anodes.

The major goal of this work was to analyse the grain size and orientation of electrodeposited alkali metal films in RFCs as well as to image pore formation after electrodissolution—in representative cross-sections—to advance the understanding of microstructure–property relations. However, before the results could be interpreted reliably, a protocol is required that does not alter the metal grain size, including artefacts created by heating during preparation and subsequent annealing. Furthermore, it was necessary to determine if and to what extent lithium and sodium anneal/recrystallize at room temperature. The following section answers these questions, while the subsequent section showcases the first results obtained from electrodeposited films within RFCs.

## Protocol for alkali metal microstructure analysis by EBSD

Lithium foils with varying microstructures were already prepared by Singh et al.^21,22^. However, while electrodeposited silver and copper films show microstructural changes during room temperature storage^24,34^, it is unclear if this can be applied to (electrodeposited) lithium and sodium. Therefore, four different metal foils were prepared for analysis as described in Methods.

**Fig. 1:**
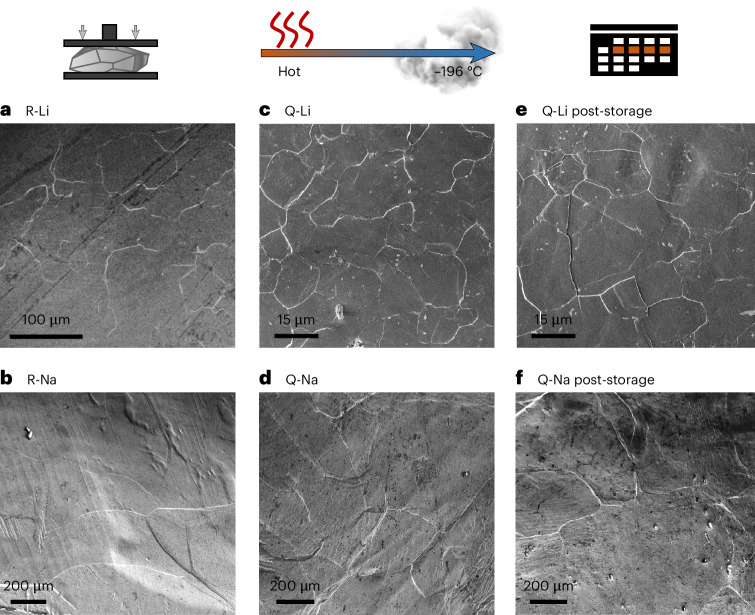
Top-view SEM images obtained from freshly pressed alkali metal foils. R-Li and R-Na denote pristine lithium and sodium foils, while quenched metal foils are labelled as Q-Li and Q-Na. **a**–**d**, Large grains are visible for lithium (**a**) and sodium (**b**) as well as small grains for lithium (**c**) and sodium (**d**). **e**,**f**, After a wait time of several calendar days, two new foils were prepared from the quenched metal, for lithium (**e**) and sodium (**f**).

Top-view scanning electron microscopy (SEM) images of each foil obtained directly after preparation are shown in Fig. 1a–d. Figure 1e,f shows top-view images of quenched lithium and sodium referred to as Q-Li and Q-Na after several days of room temperature storage, to reveal information about possible grain growth. First, lines and multiple triple junctions are found on the freshly prepared metal foils. These are assumed to be grain boundaries made visible by preferential degradation or morphological changes induced during pressing^21,22^. Interestingly, sodium foils show less pronounced lines, possibly due to differences in mechanical properties, impurity level and surface chemistry.

With apparent grain sizes of ~100–300 µm for the reference lithium foil (R-Li) and 10–50 µm for Q-Li, we confirm that thermal processing strongly influences the lithium microstructure^21,22^. Furthermore, no visible grain growth occurred during room temperature storage of Q-Li despite its high *T*_H_. With less pronounced white lines, the reference sodium foil (R-Na) shows larger grains close to the millimetre range and Q-Na grain sizes of about 200–600 µm. Note that the grains were sometimes larger than the SEM field of view, which makes statistical grain size analysis difficult. However, upon comparison, it is also striking that the microstructure of sodium could be altered by thermal processing. Furthermore, Q-Na stored for 2 days does not show grain growth caused by room temperature annealing, which is confirmed below.

The apparent lack of grain growth despite the high *T*_H_ may be explained considering two stages. Small grains are expected to grow rapidly driven by the reduction of interfacial grain boundary energies, as, for example, in the case of electrodeposited silver^25^. However, with increasing grain size, the driving force to mitigate interfacial grain boundary energies decreases, slowing down further growth.

**Fig. 2:**
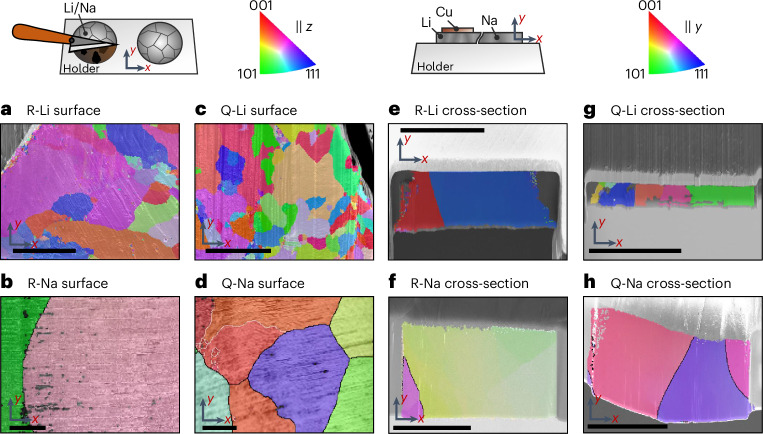
IPF maps of alkali metal foils with different thermal processing histories. **a**–**d**, Top-view IPF maps of freshly cut samples of R-Li (**a**), R-Na (**b**), Q-Li (**c**) and Q-Na (**d**). **e**–**h**, IPF maps of cross-sections prepared via cryogenic FIB of R-Li (**e**), R-Na (**f**), Q-Li (**g**) and Q-Na (**h**). Scale bars, 200 µm.

To validate these initial findings, we used EBSD to analyse freshly prepared surfaces of each foil as depicted in Fig. 2a–d. Exemplary Kikuchi patterns are depicted in Supplementary Figs. 1 and 2, proving that crystalline, sufficiently passivation-free surfaces were obtained. The inverse pole figure (IPF) maps confirm that the grains identified in Fig. 1 are body-centered cubic metal grains with different orientations. Due to the large grain size of R- and Q-Na, several spots were characterized by EBSD as presented in Supplementary Figs. 3 and 4. Similar grain sizes are observed for R-Li and R-Na with EBSD characterization compared with the initial SEM analysis as well as for their quenched counterparts. This controlled grain size change fits well to what was observed for lithium^21,22^ and has not been previously shown for sodium. As the time between quenching the metal and cooling for analysis was minimized (that is, 20 min for Q-Na), substantial grain growth is not expected to have occurred. Moreover, no substantial grain growth of Q-Na at room temperature was observed after approximately 2 weeks, as demonstrated by the IPF in Supplementary Fig. 4. Therefore, grain growth of lithium at room temperature is also unlikely due to its lower *T*_H_. Interestingly, the lithium grains do not follow a typical Voronoi shape. A more regular grain shape is expected, as seen for sodium. We assume that these shape distortions were induced by the high ductility of the metal during preparation, where a blade was run across the sample surface.

These results for thermally prepared alkali metals are consistent and demonstrate the efficacy of our protocol. However, electrodeposited metal films in RFCs are buried between a CC and an SE separator, and surface EBSD analysis is not suitable for these films. Here, cross-section preparation by cryogenic FIB is required to enable the analysis of electrodeposited metal layers perpendicular to the interface. Therefore, a second step was necessary to exclude local alterations of the microstructure by the cryogenic FIB preparation. Hence, cross-sections of samples shown in Fig. 2a–d were analysed using EBSD (Fig. 2e–h). A second IPF map in the *x* direction of the sample in Fig. 2g is shown in Supplementary Fig. 5, to showcase that the large green grain actually consists of multiple, coincidentally oriented similarly in the *y* direction. IPF maps of aged sodium cross-sections are depicted in Supplementary Fig. 6.

Although the analysis area of a cross-section is smaller than a surface view, substantial differences between thermally processed metal foils and reference foils are observed for both lithium and sodium, consistent with previous results. Furthermore, all cross-sectional maps predominantly show vertical grain boundaries. This probably occurs due to the high aspect ratio of the analysed foils and the grain size being larger than the foil thickness or potentially during texturing when pressing ingots to a foil. This also explains why more curved grain boundaries are observed for thicker sodium foils in Fig. 2f,h with a lower aspect ratio.

Local grain growth during cryogenic FIB preparation is thereby excluded, and the alkali metal microstructure remains unaltered. These findings are supported by the lack of microstructural changes after a second milling step of a cross-section (Supplementary Fig. 7). Overall, this confirms that our cryogenic FIB protocol is suitable to analyse the microstructure of alkali metal cross-sections, including electrodeposited films at CC|SE interfaces. We could further confirm that the white lines on the surfaces as seen by SEM in Fig. 1 indicate grain boundaries in most cases, as elaborated in Supplementary Fig. 8.

## The microstructure of electrodeposited alkali metal films

**Fig. 3:**
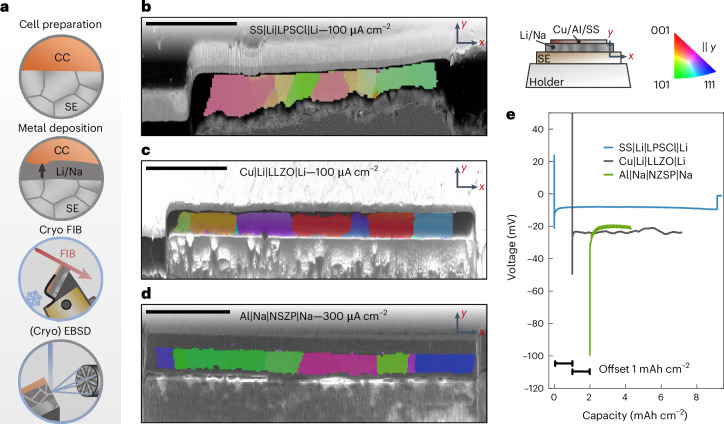
Microstructural analysis of electrodeposited lithium and sodium at different CC|SE interfaces using FIB cross-sections and EBSD. **a**, Overview of the analysis protocol to image the microstructure of electrodeposited alkali metal films. **b**,**c**, Cross-sectional IPF maps of lithium plated at the SS|LPSCl (**b**) and Cu|LLZO (**c**) interface. **d**, A cross-sectional IPF map of sodium plated at the Al|NZSP interface. IPF maps are given parallel to the *y* direction as indicated by the sketch in the upper right of the figure. Scale bars, 100 µm. **e**, The corresponding voltage profiles during electrodeposition for every layer.

Next, the EBSD analysis is focused on the microstructure of alkali metal films electrodeposited within RFCs. Different RFCs were prepared, namely, SS|LPSCl|Li, Cu|LLZO|Li and Al|NZSP|Na cells, representing today’s most investigated SEs paired with alkali metal anodes. The metal was deposited within each cell on the respective CC. Figure 3a shows the protocol needed to obtain cross-sectional EBSD images thereof, with the three respective IPF maps shown in Fig. 3b–d. The corresponding voltage profiles during plating are shown in Fig. 3e.

Both lithium films were deposited with 100 µA cm^−2^, at the SS|LPSCl interface with 15 MPa and at the Cu|LLZO interface with 5 MPa. Sodium was electrodeposited at the carbon-coated Al|NZSP interface with 300 µA cm^−2^ at 3 MPa. These parameters were chosen on the basis of experience to yield a homogeneously deposited film^11,35^. Impedance spectra before and after electrodeposition of each cell are depicted in Supplementary Fig. 9, showing a characteristic change from a blocking impedance of the working electrode to the signature of a reversible alkali metal electrode, confirming nucleation and subsequent growth of a metal layer^11,35,36^. All three voltage profiles display a characteristic nucleation overpotential in line with previous results, followed by a stable plateau, during which layer growth occurs^11,35^. The magnitude of the overvoltage is also similar to what was previously reported with 10–20 mV for lithium deposition and around 80 mV for sodium deposition^35,36^. For lithium plated at the SS|LPSCl interface, a sudden voltage drop indicates a short circuit from dendrite formation, confirmed by impedance data showing substantially lowered resistance (Supplementary Fig. 9). However, the resulting film could still be analysed.

Subsequently, each sample was prepared for EBSD analysis according to the description given in Methods. It is striking that the average grain size of each electrodeposited metal film is quite large, especially compared with other electrodeposited metals^25,28,34,37^. The grain width for deposited lithium at the SS|LPSCl interface and CC|LLZO interface is around 20–100 µm and 10–100 µm, respectively. Sodium deposited at the carbon-coated Al|NZSP interface shows a grain width around 10–150 µm. A second cross-section of the sodium film in Supplementary Fig. 10 confirms the given grain size. However, compared with the alkali metal foils analysed in Figs. 1 and 2, the grain size is smaller, further indicating the absence of substantial room temperature storage grain growth in electrodeposited films, although the impurity content is expected to be lower.

Another observation is that all grain boundaries are perpendicular to the CC|SE interface, which marks a major difference between the Li/Na|SE interface of an electrodeposited metal compared with an as-built Li/Na|SE interface using foils. In the latter case, grains are larger and grain boundaries are randomly oriented. This has implications for the subsequent discharge performance of the anode, as the microstructure will probably affect the pore formation^21,22^. Additionally, for the lithium film plated at the Cu|LLZO interface, two small grains are observed that do not span the whole thickness of the film. A larger magnification of these areas is visible in Supplementary Fig. 11.

Interestingly, similar predominant columnar grain growth has also been observed for electrodeposited nickel using cross-sectional EBSD analysis, although these grains did not span the whole thickness^37^. While the grain size is nearly constant for nickel films, the fraction of columnar grains increases with thickness. A similar phenomenon is not observed here, as the columnar grains appear to grow along the whole layer. On the contrary, electrodeposited silver films do not show this columnar grain growth^24^. A reason could be that silver shows room temperature grain annealing within hours, thereby changing the initial grain microstructure present during electrodeposition. This supports the conclusion that plated alkali metals do not show grain growth at room temperature, unlike silver and copper. However, microstructural changes during deposition may still occur, as discussed later. A direct correlation between CC/SE microstructure and the alkali metal microstructure cannot be observed, as discussed in Supplementary Figs. 12 and 13.

**Fig. 4:**
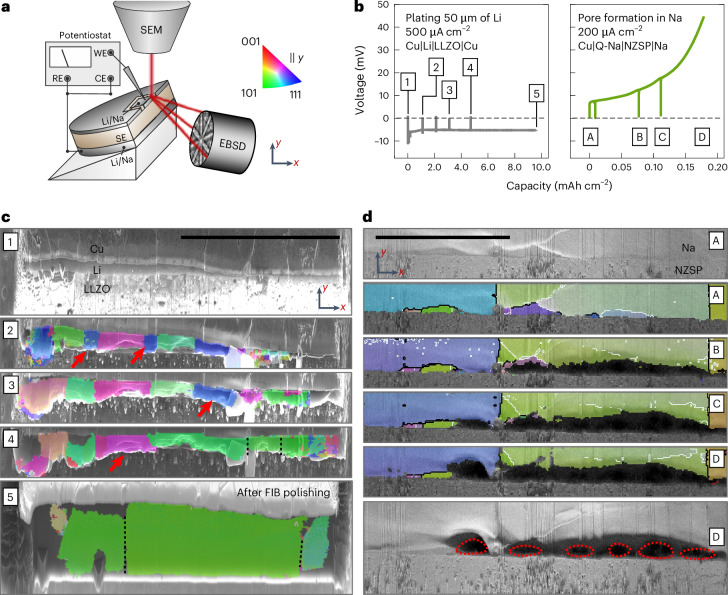
Analysis of the microstructural evolution during electrodeposition and electrodissolution of an alkali metal electrode in contact with an SE. **a**, Schematic depiction of the in situ EBSD setup whereby RE, WE and CE denote the reference, working and counter electrode, respectively. **b**, The voltage profiles for plating 50 µm lithium at the Cu|Li|LLZO interface and stripping sodium until pore formation at the Cu|Q-Na|NZSP interface. **c**,**d**, The microstructure evolution for lithium plating (**c**) and sodium stripping (**d**). The red arrows indicate grains disappearing during film growth in **c**, and the red outlines indicate pores formed during stripping in **d**. The map provided in **c** step 5 was acquired after a 2 week storage period and second FIB polishing step. Scale bars, 100 µm.

As the process of grain growth and evolution during electrodeposition is yet elusive, in situ EBSD analysis was performed. Here, cross-sections of Cu|Li|LLZO|Li and Cu|Q-Na|NZSP|Na were charged and discharged inside the SEM, respectively, and stopped for intermittent EBSD analysis. Around 2 µm of lithium was deposited at the Cu|LLZO interface before cross-section preparation to fix the CC on the SE. Further, microelectrodes were prepared via FIB at the cross-section to ensure that changes occur in the field of view. The described setup is depicted in Fig. 4a. An overview SEM image is shown in Supplementary Fig. 14a before deposition with front-view images of the pristine cross-section provided in Supplementary Fig. 14b,c. The left section of Fig. 4b shows the voltage profile during deposition of lithium with 1,000 µA cm^−2^ initially followed by ~500 µA cm^−2^. The initial higher voltage ensures homogeneous layer growth. The observed voltage profile is mostly flat, hinting at dendrite-free lithium deposition of 10 mAh cm^−2^, corresponding to a layer of ~50 µm thickness. The right side of Fig. 4b shows the voltage profile obtained for stripping of the sodium electrode with a typical increase of voltage indicative of pore formation. Both measurements were paused for intermittent EBSD analysis as visible in the potential profiles, which is shown in Fig. 4c,d.

Figure 4c(1) shows an SEM image of the pristine cross-section of Cu|Li|LLZO. The lithium reservoir is not freshly deposited and also too thin to obtain EBSPs of sufficient quality to generate an IPF map. However, upon depositing around 10–15 µm of additional lithium, the IPF map depicted in Fig. 4c(2) was obtained. Herein, several grains around 10–30 µm wide are visible. Surprisingly, after another ~5 µm of lithium deposition, fewer but wider grains are visible in the next IPF map in Fig. 4c(3). Two small blue grains close to a <111> orientation from the previous map apparently fused into larger neighbouring grains close to a <101> orientation (green). After another deposition step, IPF map Fig. 4c(4) shows even wider grains, with another blue grain from the previous map being fused to a neighbouring green grain.

After another long deposition step resulting in around 20 µm of additional lithium, multiple grains have fused together forming a large grain with >100 µm width, again close to a <101> orientation as visible in Fig. 4c(5). As the observed grain boundaries are mostly vertically oriented, lateral motion of grain boundaries is assumed to be responsible for the observed changes. This post-deposition map was acquired after 2 weeks of room temperature storage and additional FIB polishing, with top- and front-view images depicted in Supplementary Fig. 14g,h. Additionally, the map in Supplementary Fig. 15a acquired directly after the deposition shows the same microstructure. Small deviations were visible in the map directly acquired after plating, which can be seen in Supplementary Fig. 15a. This is due to the lithium being partly squeezed outside of the prepared electrode area (Supplementary Fig. 14d). The uneven film morphology at the interface also explains the low indexing rate of the shown IPF maps, as the sample is not perfectly tilted at 70° at every spot. Interestingly, our analysis also confirms the absence of grain growth during storage. Additionally, Supplementary Fig. 15b,c depicts the maps of Fig. 4c(4,5) with the IPF in the *x* direction, showing that the large green grains actually consist of more grains, coincidentally oriented similarly in the *y* direction. These grain boundaries are indicated with dashed black lines in every map in the *y* direction. Furthermore, Supplementary Fig. 15d depicts the cross-section shown in Fig. 4c(5) after storage and additional FIB polishing, further confirming the absence of grain growth during storage. We consider these results gained from the in situ analysis as highly important, that is, microstructural changes are now shown to occur during the deposition process but to stop once the deposition is finished. This is also explored for sodium metal deposition as discussed in Supplementary Fig. 16.

To further study the dependence of pore formation on the anode microstructure, a sodium anode was stripped with intermittent EBSD analysis. A typical voltage profile is achieved with an initial small increase evolving into a step increase, as shown in Fig. 4b. This signature voltage profile clearly indicates pore formation^6,38–40^. The corresponding IPF maps and forward-scatter electron images are shown in Fig. 4d. Starting from an optimal interfacial contact between sodium and NZSP (Fig. 4d(A)), a dark region close to the interface emerges (Fig. 4d(B)) after the first stripping interval, attributed to pores. Likewise, the generation of secondary electrons decreases, leading to darker areas in the images (Supplementary Fig. 17a,b).

Interestingly, the pores formed mainly within the green grain, while the vertical grain boundaries on both sides remain intact (Fig. 4d(C)). As stripping progresses, another pore nucleates within the large blue grain starting from a grain boundary, supporting the previous description. Moreover, the pores within the green grain further grow into its bulk (Fig. 4d(D)). Despite geometrical correction, the size and shape of the pores might not be fully captured, appearing larger due to the high tilting angle of 70° for EBSD analysis, as visible in Supplementary Fig. 17c. Finally, the cross-section was polished again to confirm that the pores resulted from anodic dissolution of sodium (Supplementary Fig. 17d).

Clearly, this observation of preferential pore formation in grains—not in grain boundaries—is at first glance counter-intuitive, but is a direct proof for the fast diffusion of vacancies along grain boundaries^41^. Pore nuclei at grain boundaries can be closed faster by diffusion within the grain boundary than within the bulk. The microstructure strongly influences the pore formation and, thus, the physical contact at the interface, which drastically affects the performance of the metal anode.

## Grain evolution during cycling

**Fig. 5:**
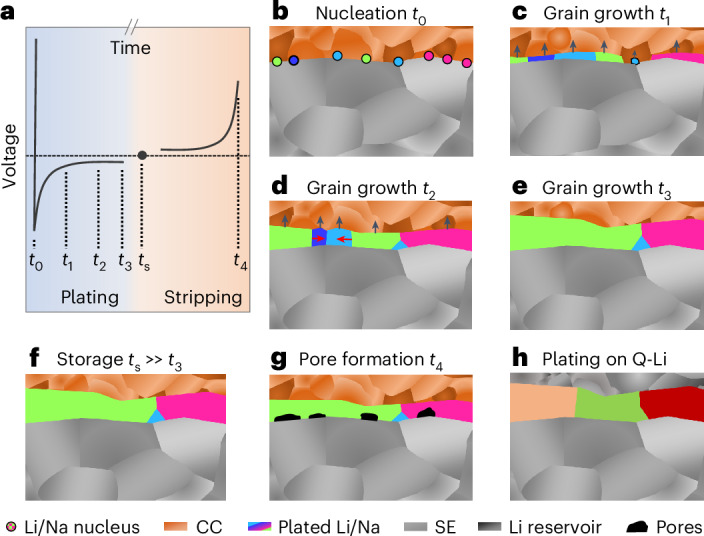
Schematic evolution and the origin of the observed columnar microstructure of deposited alkali metal films in RFCs during electrochemical deposition and dissolution. **a**–**g**, A schematic voltage profile of one cycle of a metal electrode (**a**) with the corresponding schematized metal microstructures upon nucleation (**b**) followed by grain growth at *t*_1_ (**c**), *t*_2_ (**d**) and *t*_3_ (**e**), after storage (**f**) and after pore formation induced by electrochemical dissolution (**g**). **h**, The schema of the metal microstructure plated on a lithium reservoir.

In the following, a mechanism behind the microstructural evolution during stripping and plating of alkali metal is proposed and schematically depicted in Fig. 5.

Figure 5a shows a schematic of a voltage profile during electrochemical deposition of alkali metals on an inert CC with subsequent storage and dissolution. The metal nucleation at *t*_0_ evolves into the early stages of grain formation and growth (Fig. 5b), which is followed by mostly vertical grain growth at *t*_1_ with different rates (Fig. 5c,d). If the growth rates of neighbouring grains are dissimilar, it also seems to be possible that the growth of slower growing grains is limited compared with the faster-growing neighbouring grains as shown in Fig. 5d,e or Supplementary Fig. 11, leading to truncated V-shaped grains. Furthermore, the observed grain density is lower than what would be expected for each nucleus growing into a columnar grain^12,13^. One reason is that neighbouring nuclei may exhibit a similar orientation, thus directly fusing together to form a single grain upon contact as depicted with the pink nuclei in Fig. 5b,c.

During the whole deposition process between *t*_0_ and *t*_3_, lateral grain ripening occurs with some grain boundaries moving into adjacent grains as depicted with red arrows in Fig. 5d, thus widening the average lateral grain dimensions and reducing the grain density. The reason and magnitude of this ripening is yet elusive and may be related to abnormal grain growth or ‘secondary recrystallization’, which is dependent on the interface and grain boundary energies^42^. This may also be another reason for the occurrence of truncated V-shaped grains. To elucidate this, further studies will focus on more detailed investigations of the current density and thickness dependence of the film microstructure.

After a prolonged room temperature storage at *t*_s_, the microstructure remains unchanged (Fig. 5f). If this microstructure is then subjected to anodic dissolution until pore formation, the pores predominantly form within grains at the interface and not at grain boundaries, where vacancy accumulation is less likely during stripping (Fig. 5g). Grain boundaries as two-dimensional defects typically have a higher free energy compared with the bulk, meaning that the oxidation of a metal atom, and thus vacancy injection, is thermodynamically favoured. However, fast transport of vacancies along grain boundaries—as reported as a result of computational work by Yoon et al.—might be the reason for a kinetic stabilization of the interface, as vacancy accumulation is hindered^41^. Therefore, the growth of pores where the grain boundaries within the metal meet the interface is suppressed.

As the pores formed during anodic dissolution seem to form preferentially within grains and not at grain boundaries, we conclude that it is possible to tune the electrochemical properties, such as available stripping capacity^38,43^, by controlling the microstructure. Following this reasoning, achieving a microstructure with small grains would be desired^41^. The nucleation density mainly depends on applied current density and temperature as well as the surface properties of the SE^12,13,36^. Additionally, our in situ EBSD results further show that not only the grain nucleation is important to control the resulting microstructure, but also the growth process. Potentially, the applied current density will also influence the grain ripening during electrodeposition. Similarly, applying stack pressure may be a suitable tool to guide the lateral expansion of the growing grains^11^.

In addition, based on our evidence, controlling the microstructure of the CC and SE may not be a successful path to influence the deposited metal microstructure. Lithium plated on a lithium foil matches neither the microstructure of the SE nor the foil but plates with a similar, columnar shape as in the case of plating on an inert CC in a reservoir-free setup (Fig. 5h). The microstructure of the deposited metal is still substantially different and has lower overall grain sizes compared with those of commonly used metal foils.

Based on our results, the merging of grains is caused by the movement of grain boundaries. Thus, the microstructure of electrodeposited alkali metals is tunable by modifying the mobility and movement of grain boundaries. The mobility of grain boundaries in metals is diminished by impurities, even at low concentrations^44^. Therefore, the implementation of tailored impurities such as seed layers or particles at the metal|SE interface could affect the mobility of grain boundaries and, thus, the resulting microstructure. For example, an enhanced stripping performance for electrodeposited lithium was observed when gold particles were situated at the Cu|LLZO interface during the initial electrodeposition of lithium^14^, potentially leading to smaller grains. The mobility of grain boundaries is also prevented by introducing mechanical barriers, such as geometric constrictions present in three-dimensional SE host structures, as demonstrated for LLZO and NZSP by Wachsman and coworkers^45,46^. Our analyses on the metal microstructure offer valuable insights to explain the mechanism behind the metal growth when using three-dimensional hosts or seed layers previously overlooked in literature.

The results of this work highly motivate further research on the influence of deposition parameters and seed layer concepts or geometrical confinements at the interface on the resulting electrode microstructure and thereby subsequent electrochemical performance. Both ex situ and in situ experiments will help to develop more advanced plating and stripping strategies for enhanced performance.

We developed an analysis protocol to reliably analyse the microstructure of alkali metal foils both from the surface and cross-sections using EBSD. We conclude that the microstructure of both lithium and sodium can be tailored by thermal processing, yielding grain sizes between 10–200 µm (lithium) and 200–600 µm (sodium). Grain growth can be ruled out for these foils during both room temperature storage and cryogenic FIB preparation.

This further allowed the analysis of the grain size and orientation of electrodeposited metal for three different CC|SE combinations, namely, Cu|LLZO, SS|LPSCl and Al|NZSP. We show that the grain width in electrodeposited films in RFCs ranged between 10 µm and 100 µm while the height depends on the film thickness (‘row of teeth’ microstructure). Grain boundaries are predominantly perpendicular to the CC|SE interface, which is distinctively different from the microstructure of metal foils and thereby will influence the electrochemical performance. Interestingly, microstructural changes could be observed neither for deposited lithium nor sodium during storage. Moreover, the deposited metal microstructures were influenced neither by the SE nor the CC. Instead, we conclude the deposited metal microstructure is dominated by the used charging protocol, that is, current density and especially the capacity (layer thickness) and applied pressure—which opens a wide range of opportunities for optimization in practice.

In situ EBSD analysis during cross-sectional deposition and dissolution further revealed the evolution of the microstructure, where small grains are annealed in a process similar to Ostwald ripening in the case of lithium. Additionally, it was shown that pore formation during discharge of the metal anode predominantly occurs at the interface between the bulk of the grain and the SE. The locations where the metal grain boundaries meet the interface are mostly left intact, probably due to faster metal and vacancy diffusion along grain boundaries.

In general, this analysis advances the investigation of metal electrodes and the understanding of their microstructural evolution. A reliable analysis protocol is presented to characterize the microstructure of deposited lithium or sodium, and the presented results will help to optimize alkali metal electrodes to tune their electrochemical performance.

## Methods

### Materials

Lithium (99.0%) from Goodfellow GmbH and sodium metal supplied by BASF AG were used without further purification for microstructural characterization by EBSD, referred to as R-Li and R-Na. Modification of the microstructure of the alkali metals was induced by melting mechanically cleaned lithium and sodium ingots in stainless-steel crucibles on a hot plate at 400 °C and 250 °C, respectively, followed by quenching in liquid nitrogen inside an argon-filled glovebox. Quenched lithium and sodium are referred to as Q-Li and Q-Na. All visible passivation layers on the metal ingots were mechanical removed. Cross-sections for EBSD analysis were prepared by cutting through an ingot using a microtome blade. To avoid a potential passivation layer ‘freezing’ the microstructure in place, a fresh foil was prepared from the same quenched ingot after storage.

Counter electrodes attached to LLZO were prepared from a 750 µm lithium foil supplied by Alfa Aesar. For argyrodite-based cells, counter electrodes were prepared from a lithium rod (Albemarle Corporation). The passivation layers on the alkali metals were scraped off before usage. Three different kinds of SE were used. The LLZO was prepared as described in the literature^47^, while the LPSCl was a commercial powder by NEI with a particle size of <1 µm. NZSP was synthesized following literature methods^48^. An excess of 4.5% of the sodium precursor and 1.5% of phosphorus precursor was used for the synthesis^49^ yielding NZSP pellets with a relative density ≥90% after sintering.

### Electrode preparation and electrochemistry

Electrodeposition of lithium and sodium were carried out using three different cell configurations, namely, Cu|LLZO|Li, SS|LPSCl|Li and Al|NZSP|Na. For the garnet-based cells, copper foil (10 µm) was hot-pressed onto the polished pellets at 900 °C in accord with previous literature^11^. In preparation for the in situ EBSD measurement, nominally 2 µm of lithium was plated ex situ at 60 °C under 2.5 MPa stack pressure and current density of 250 µA cm^−2^. For argyrodite-based cells, LPSCl powder was pressed on stainless-steel foil (20 µm, Goodfellow, AISI 304) at 400 MPa. For the sodium-based RFC configuration, an aluminium sheet was coated with a thin carbon layer via tape casting: namely, carbon black (FW200, Degussa) and poly(vinylidene) fluoride were dispersed in *N*-methyl-2-pyrrolidone with a weight ratio of 95:5 carbon:poly(vinylidene) fluoride. The slurry was rapidly cast onto an aluminium foil (~17 µm) at a thickness of 30 µm at 60 °C followed by a drying process at 80 °C in vacuum for 12 h. Cylindrical electrodes were punched out and isostatically pressed onto a dry polished NZSP pellet (P1000 SiC grinding paper (Buehler)) at 100 MPa for 15 min, resulting in a 1-µm-thick carbon layer between the aluminium foil and NZSP. A sodium counter electrode was prepared as described in a previous report and attached on the opposite side of the NZSP pellet^6^. To ensure a homogeneous pressure distribution of 3 MPa during electrodeposition, the aluminium CC was covered with a nickel disc (thickness ~1 mm) that was polished with an AutoMet300 polishing machine (Buehler) using a 1 µm polycrystalline diamond suspension (MetaDi Supreme, Buehler). The obtained stack was sealed in a pouch bag under vacuum.

Electrochemical deposition was carried out using a potentiostat (Biologic, VMP300) at 25 °C if not specified otherwise in the text and controlled by EC-Lab (V11.2). Impedance measurements were carried out between 7 MHz and 100 mHz if not specified otherwise, with an excitation voltage of 10 mV. The stack pressure during deposition was controlled using an in-house built pressure frame^38^. Connection of the electrode inside the SEM was enabled using a micromanipulator system (Kleindiek Nanotechnik GmbH). In situ EBSD experiments were conducted using an SP200 potentiostat (Biologic) equipped with a low current module.

### Cross-section preparation via FIB and TIC and transfer

Small lithium and sodium metal ingots were inserted into a homemade holder tilted at a 70° angle to the horizontal. The metal surface was prepared by cutting it with a microtome blade along the holder at a 70° angle, which is optimal for EBSD analysis, resulting in a surface that was free enough from any passivation to enable the EBSD analysis. For cross-sectional EBSD analysis, passivation-free ingots of lithium and sodium were pressed into metal foils and mounted on a home-built holder with a tilt angle of 20° to the horizontal. Cross-sections perpendicular to the metal surface were processed using a plasma FIB (XEIA3 system, Tescan GmbH). Milling was conducted under cryogenic conditions (−130 °C) using Xe^+^ ions operating at 30 kV with beam currents between 0.1 µA and 2.7 µA. To characterize the electrodeposited lithium and sodium at the CC|SE interface, the assembled Al|Na|NZSP|Na and Cu|Li|LLZO|Li cells were intentionally fractured perpendicular to the sample surface. The fracturing was carried out by using two flat and insulated pliers. The distance between the pliers was kept at several millimetres so as not to press onto the deposited metal beneath the CC near the desired fracture line. Furthermore, the samples were always broken with the electrode of interest being subjected to tensile forces during the fracture. In the case of the SS|Li|LPSCl|Li cells, the CC including the deposited lithium was peeled off from the SE and cut using scissors instead. The cross-sections were further processed according to the previously described plasma FIB approach to achieve clean cross-sections for EBSD mapping. Alternatively, large-area cross-sections for EBSD maps were also prepared at −130 °C with a triple ion beam cutter (EM TIC 3X, Leica Microsystems) equipped with three Ar^+^-ion guns operating at 6 kV and a current of around 2 mA instead of the FIB.

All sodium samples were transferred under inert gas and cryogenic conditions (−160 °C) between the glovebox and the respective FIB-SEM and high-resolution-SEM using a Leica EM VCT500 transfer system (Leica Microsystems). Lithium samples were transferred under cryogenic conditions from the time of interface preparation via FIB and onwards.

### In situ SEM and EBSD

Microstructural characterization was performed using a high-resolution field-emission SEM instrument Gemini SEM 560 (Carl Zeiss Microscopy GmbH) equipped with a Symmetry 3 EBSD detector (Oxford Instruments) operated by Aztec 6.1 software package (Oxford Instruments). EBSPs were recorded at 20 kV excitation voltage and a beam current of 3.4 nA and partially at 10 kV with 12.3 nA for lithium, if the EBSP quality allowed. Exposure time, pattern averaging, background correction and shadow masking were optimized for each sample individually to obtain optimal pattern quality. The EBSPs were indexed via a Hough algorithm with a resolution of 60 and 6–11 bands considering the following phases: cubic Na (*Im*$\bar{3}$*m*, Inorganic Crystal Structure Database (ICSD) number 44757), cubic Al (*Fm*$\bar{3}$*m*), cubic Cu (*Fm*$\bar{3}$*m*) and Li (*Im*$\bar{3}$*m*). Hexagonal Na_2_O_2_ (*P*$\bar{6}$2*m*, ICSD 26575) and cubic Na_2_O (*Fm*$\bar{3}$*m*, ICSD 60435) were only considered to validate that no misindexing occurred due to surface passivation during sample preparation and processing.

Evaluation and post-treatment of the recorded EBSD maps were performed with an AZtecCrystal software package (Oxford Instruments). Besides using a common Hough indexing algorithm, EBSPs were indexed with a dynamic simulated pattern matching algorithm to enhance the indexing rate and reduce misindexed pixels (zero solutions). Based on the crystal structure of lithium and sodium metal, EBSPs of different orientation were simulated and matched with the measured EBSP. First, the recorded EBSPs were calibrated followed by a pixel binning. For indexing, the master pattern was calculated with an orientation spacing of 2° and matched with the measured EBSP. Second, the matched pattern orientation was refined to decrease the deviation. Finally, a matched pattern of a pixel is compared with the next neighbour pixel, and zero solutions were replaced if the band contrast was higher than 10. For post-treatment of sodium-based maps, a Gaussian filter was applied on the measured EBSP to reduce the influence of partially masked EBSPs. The combination of indexing procedures is visualized in Supplementary Figs. 1 and 2.

After the dynamic pattern matching, mathematical data refinement was performed by wild spikes removal followed by replacing zero solution with six neighbours and five neighbours. Pseudo-symmetry removal of 60° <111> for Laue group *m*3*m* was only applied after verifying that the respective raw EBSPs are similar. If not otherwise stated, large angle grain boundaries are indicated by black lines with a misorientation of neighbour pixels >10°, while misorientation between 2° and 10° is highlighted by white lines for sodium.

For in situ EBSD experiments on sodium, an NZSP pellet was first cut perpendicular to the sample’s surface with a diamond saw. The cross-section was polished with diamond grinding paper using a Leica EM TXP system (Leica Microsystems). The sodium working electrode was prepared from Q-Na with a diameter of 3 mm while the counter electrode consisted of R-Na with a diameter of 8 mm. The circular electrodes were sliced with a microtome blade and positioned under a digital microscope (Emspira 3, Leica Microsystems) to match the polished cross-section of the NZSP pellet. Cross-sections for in situ EBSD experiments on lithium were prepared out by fracturing the respective Cu|Li|LLZO|Li pellet similarly to the post-mortem analysis, followed by FIB polishing and microelectrode preparation. Samples were generally handled in an argon-filled glovebox (<0.1 ppm O_2_, <1 ppm H_2_O, MBraun).

## Online content

Any methods, additional references, Nature Portfolio reporting summaries, source data, extended data, supplementary information, acknowledgements, peer review information; details of author contributions and competing interests; and statements of data and code availability are available at 10.1038/s41563-024-02006-8.

## Supplementary information

Supplementary Information Supplementary Figs. 1–17 and Discussion.

## Data Availability

The data that support the findings of this study are available at the open access repository JLUdata under 10.22029/jlupub-18714.
