# Supplementary material for: Imaging the microstructure of lithium and sodium metal in anode-free solid-state batteries using electron backscatter diffraction
Source: Nat Mater. 2024 Sep 23;23(12):1678–85. doi: 10.1038/s41563-024-02006-8 (PMC11599044; doi:10.1038/s41563-024-02006-8)
Supplement: Supplementary file 1 — Supplementary Figs. 1–17 and Discussion. [file 41563_2024_2006_MOESM1_ESM.pdf]

nature materials

Supplementary information https://doi.org/10.1038/s41563-024-02006-8

Imaging the microstructure of lithium and sodium metal in anode-free solid-state batteries using electron backscatter diffraction

In the format provided by the authors and unedited

Table of Contents

1 Kikuchi Pattern of Alkali Metals 2
2 Inverse Pole Figures of Quenched and Reference Sodium Metal 4
3 Cross-sectional Inverse Pole Figures of Alkali Metal 6
4 Impedance Spectra of Electrodeposited Alkali Metals at CC|SE Interface 10
5 Cross-sectional Inverse Pole Figures of Electrodeposited Alkali Metal 11
6 Influence of SE or CC Microstructure on Electrodeposited Metal Microstructure 13
7 In situ Lithium Electrodeposition at the Li|LLZO Interface 15
8 In situ Sodium Electrodeposition at the Q-Na|NASICON Interface 17
9 In situ Sodium Electrodissolution at the Q-Na|NASICON Interface 20
10 References 21

1 *Kikuchi Pattern of Alkali Metals*

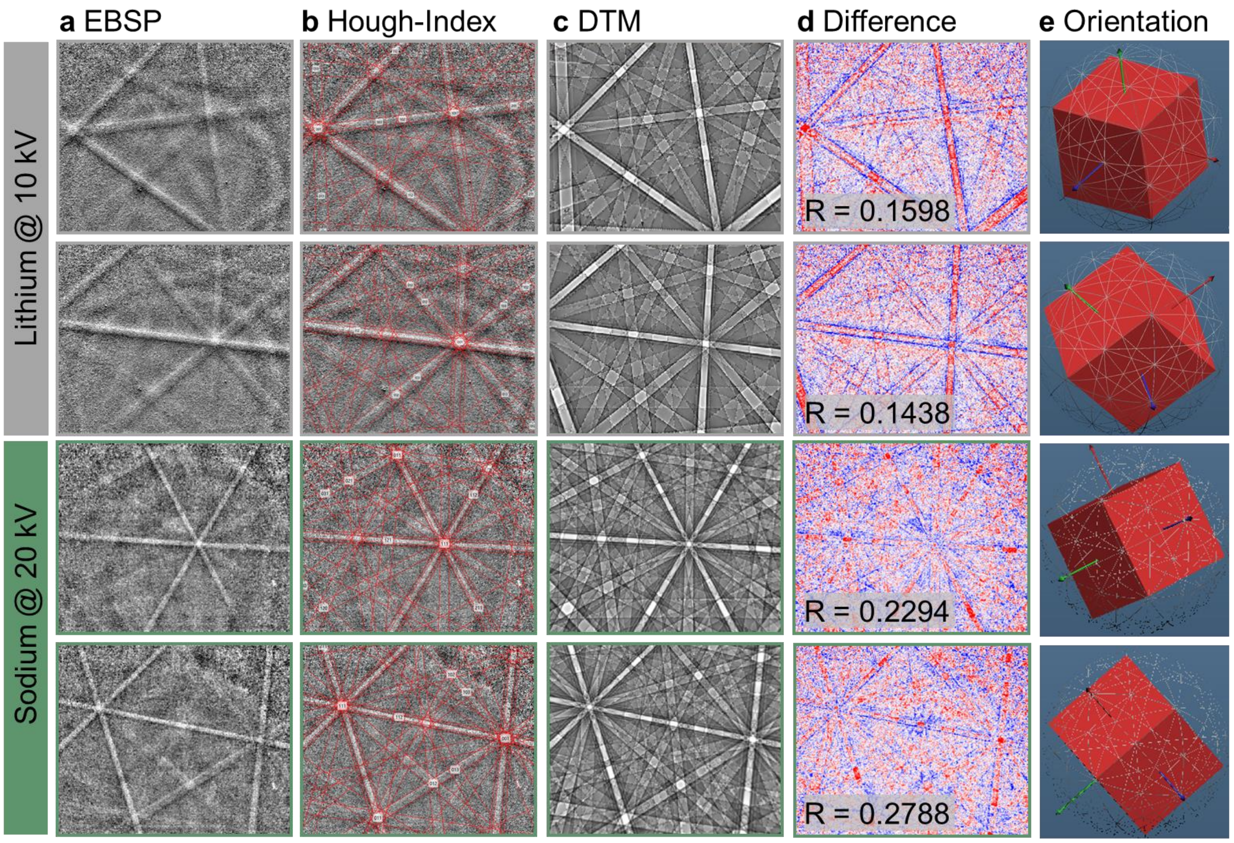

Figure S1. *a) Experimentally obtained representative electron backscatter pattern (EBSP) (Kikuchi pattern) of lithium and sodium metal at two different orientations each. The grain orientation of each pixel is determined by the respective Hough-indexing of the EBSP, as shown in b). To enhance the indexing of less resolved EBSPs and reduce the number of incorrectly indexed pixels, an additional indexing process using a dynamic simulated pattern was conducted. EBSP for different orientations were dynamically simulated based on the crystal structure of each metal and are shown in c). The measured and simulated (EBSPs) were compared until the difference between them was minimized in d). The orientation of the pixel is determined based on the match, as demonstrated in e). Lithium patterns were acquired at 10 kV, whereas sodium patterns were acquired at 20 kV. The pattern quality as well as the R-value obtained by matching the patterns with simulations is superior for sodium due to its higher material density and therefore higher intensity of backscattered electrons.*

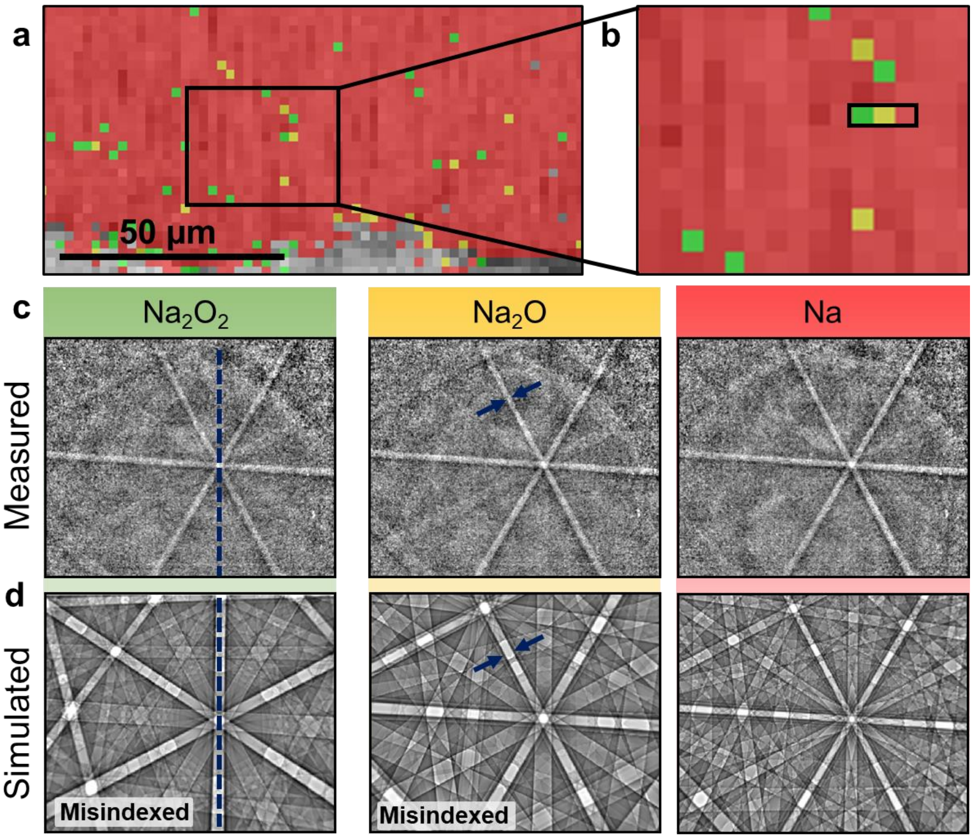

Figure S2. *a) Phase map of a representative sodium cross-section prepared via FIB-SEM without data cleaning. Three different phases were considered during the Hough-indexing algorithm, namely, Na_2O_2 (ICSD 26575), Na_2O (ICSD 60435), and Na (ICSD 44757) indicated in green, yellow and red, respectively. A magnified region of the phase map is shown in b), where a row of pixel is highlighted by a black frame. According to the phase map all phases are present next to each other although the measured EBSP patterns are the same, as visualized in c). Based on the simulated pattern for the respective phase shown in d), the Na_2O_2 and the Na_2O phases are clearly indexed incorrectly. For the Na_2O_2 phase distinct Kikuchi bands are missing (indicated by the blue dotted line), while for the Na_2O phase the band width does not match that of the measured EBSP (blue arrows). The measured EBSP can thus be assigned unequivocally to the sodium metal phase. Furthermore, the formation of oxide-based side phases resulting from residual gases in the SEM chamber can be eliminated. This supports the conclusion that the established workflow is appropriate for characterizing the microstructure of alkali metals.*

2 *Inverse Pole Figures of Quenched and Reference Sodium Metal*

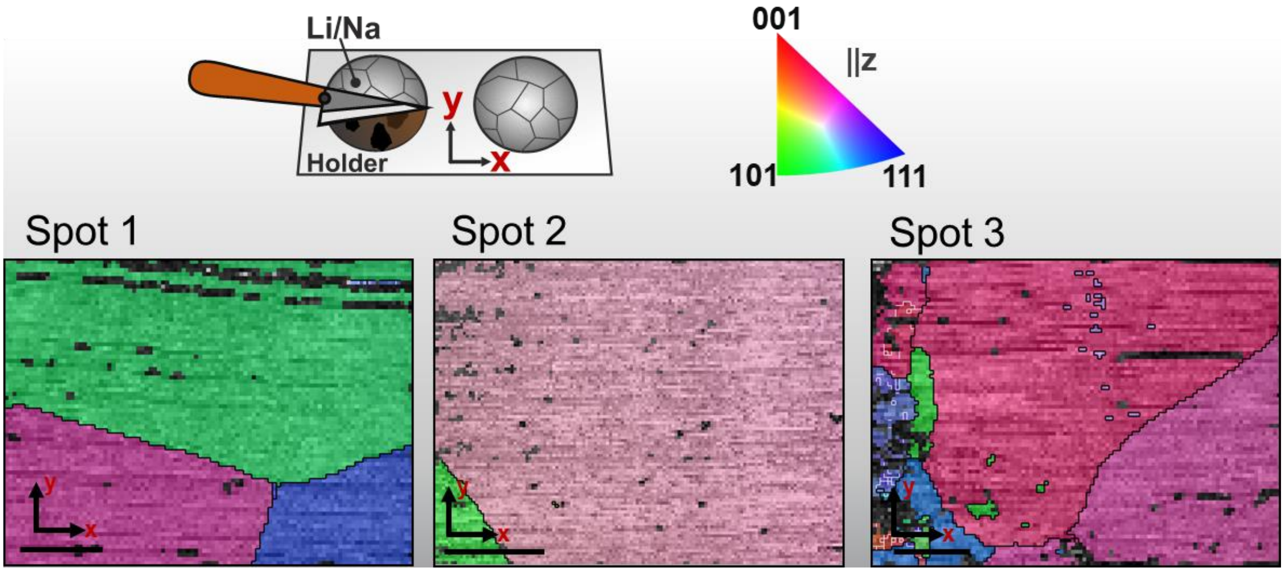

Figure S3. *The inverse pole figure (IPF) map of R-Na was obtained at various spots. The IPF is presented parallel to the z-direction (normal direction, ND), according to the sample coordination system shown in the schemes on the top right. Large angle grain boundaries (>10°) are indicated by black lines. Misorientations between neighboring pixels in the range of 2° - 10° are only observed at spot 3, as marked by white lines. The scale bar in each image equals 200 µm. Grains are observed in the range from a few hundred micrometers to almost one millimeter. Due to the huge grain size and the corresponding small number of grains in the imaged samples, no meaningful grain size distribution can be determined, although several sample spots were analyzed.*

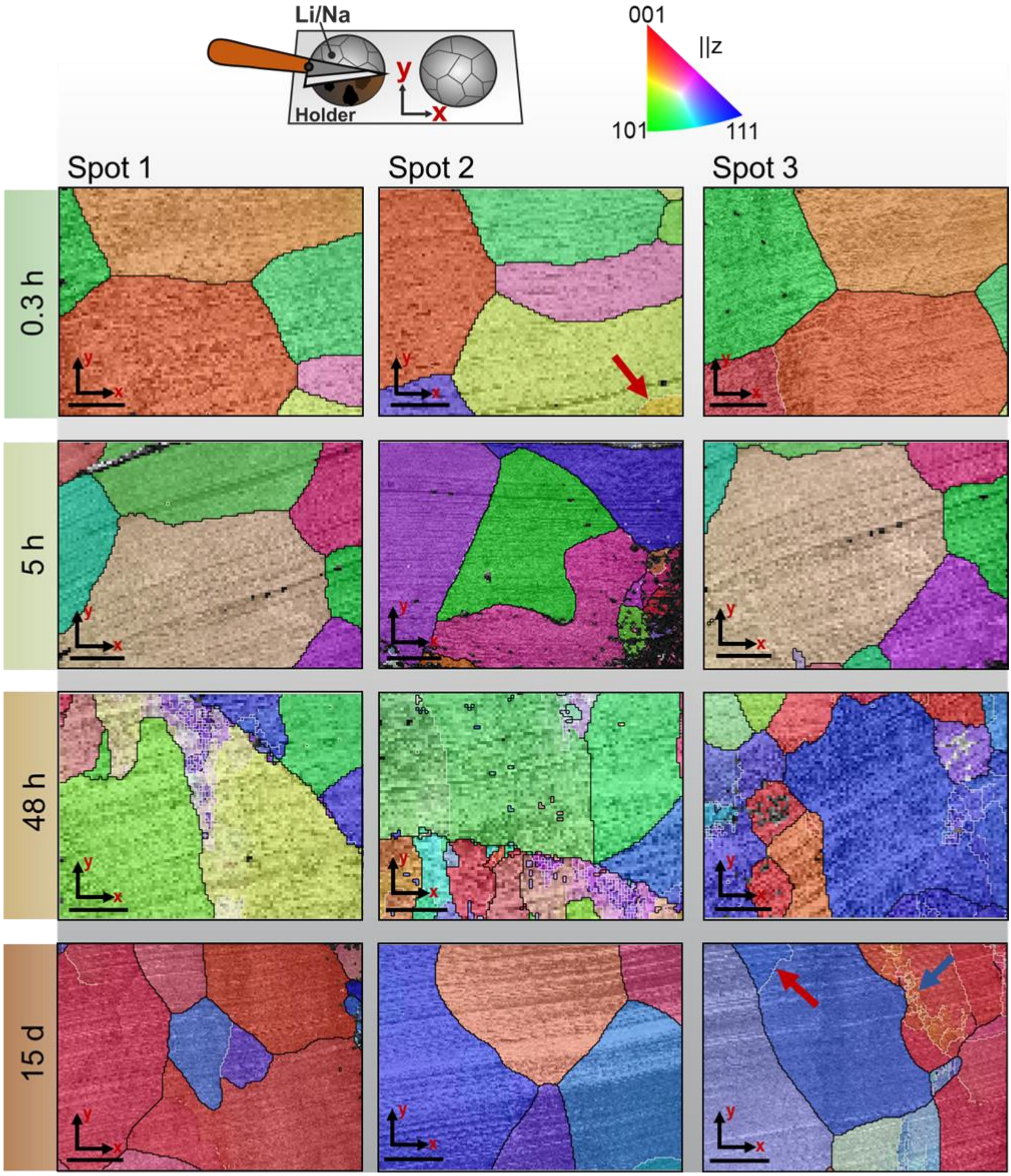

Figure S4. *Time series of inverse pole figures from Q-Na at various spots. The IPF is presented parallel to the z-direction (normal direction), according to the sample coordination system shown in the schemes on the top right. Large angle grain boundaries (>10°) are indicated by black lines. White lines indicate misorientations in between 2° - 10°. It is important to note that this angle range includes both small grain boundaries and material deformations. For instance, in the IPF map in the lower right a small angle grain boundary is indicated by a red arrow and deformed regions marked by a blue arrow. The scale bar in each image denotes 200 µm. The grain size ranges from few to several hundred microns. Although various areas of the Q-Na were captured, a reliable grain size distribution cannot be determined due to the large size and low number of grains. No grain growth is observed over time as a result of annealing.*

3 Cross-sectional Inverse Pole Figures of Alkali Metal

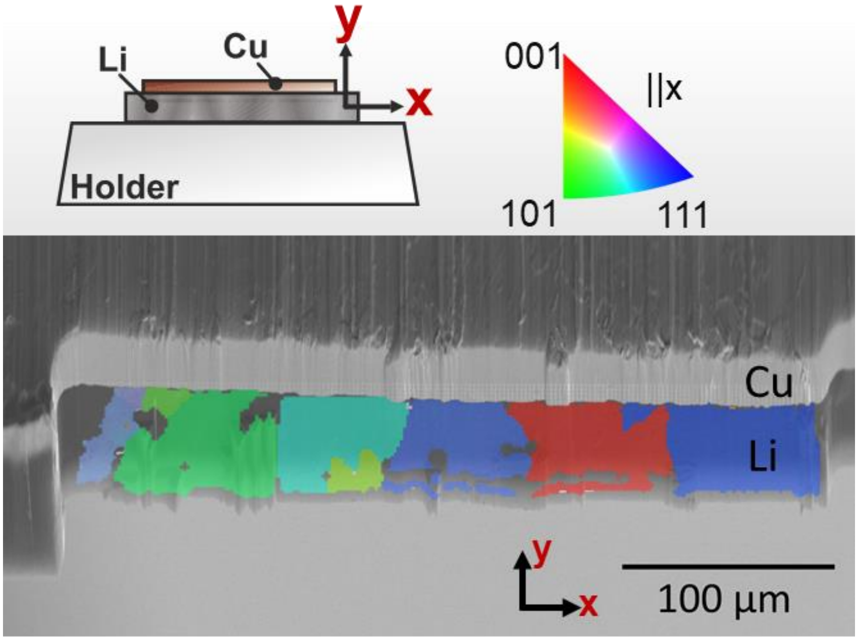

Figure S5. *Magnified IPF map of the sample shown in Figure 2g parallel to the x-direction. Clearly, the large green area assumed to be one grain parallel to the y-direction actually consists of two grains, coincidently oriented the same in y-direction but different in x-direction.*

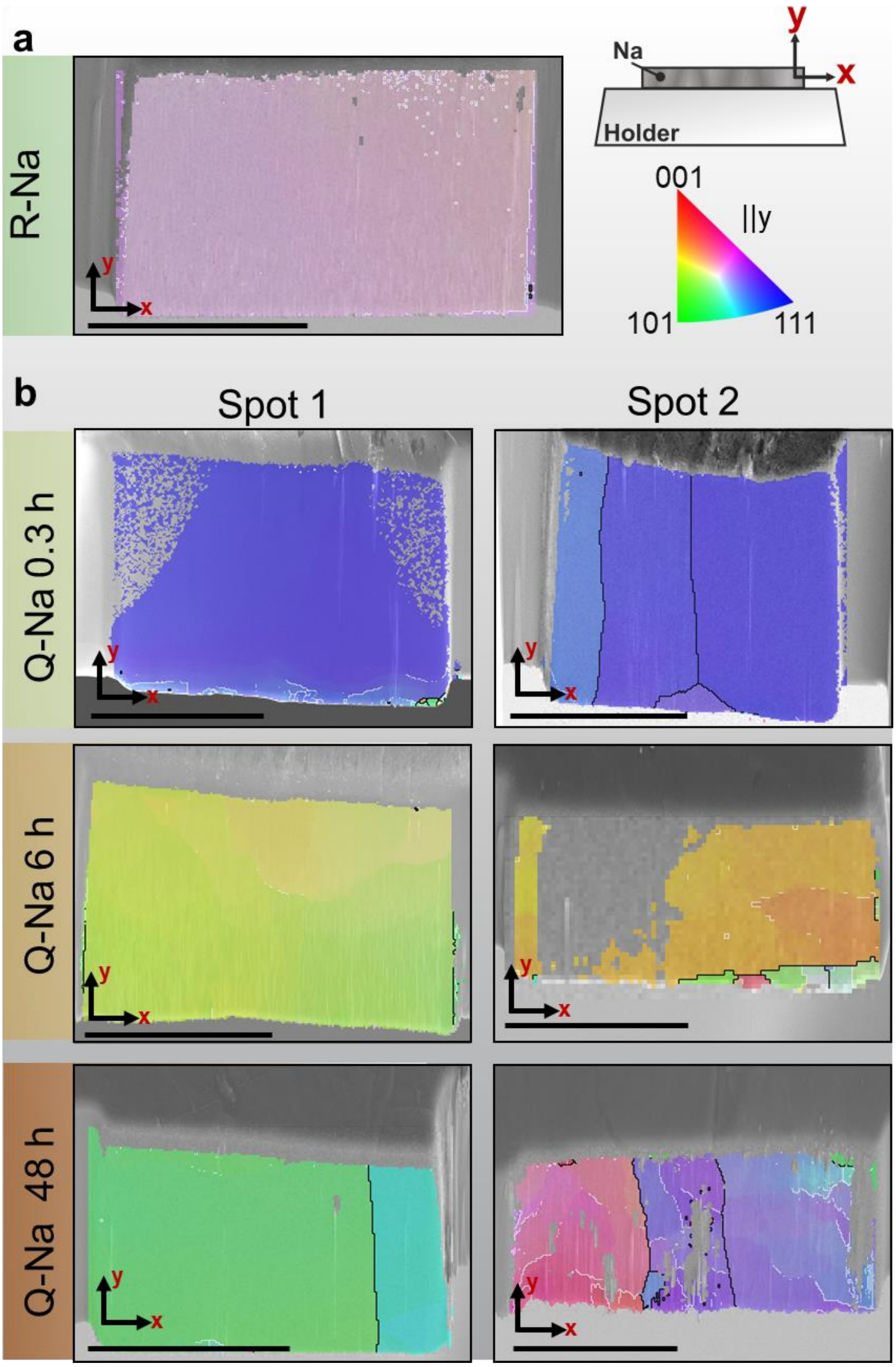

Figure S6. *Compilation of cross-sections of a) R-Na and b) Q-Na foils prepared via focused-ion beam milling. The IPF is presented parallel to the y-direction (milling direction), according to the sample coordination system shown in the schemes on the top right. Large angle grain boundaries (>10°) are indicated by black lines. Misorientation in the angle range between 2° to 10° are highlighted by white lines. The scale bar in each image equals 200 µm. Due to the large size of sodium grains, complete grains cannot be visualized in cross-sections. No opposite trend is seen in the FIB-SEM cross-sections compared to the samples prepared in* ***Figure S4****. The vertical grain boundaries also exhibit a preferred orientation, which can be attributed to the aspect ratio.*

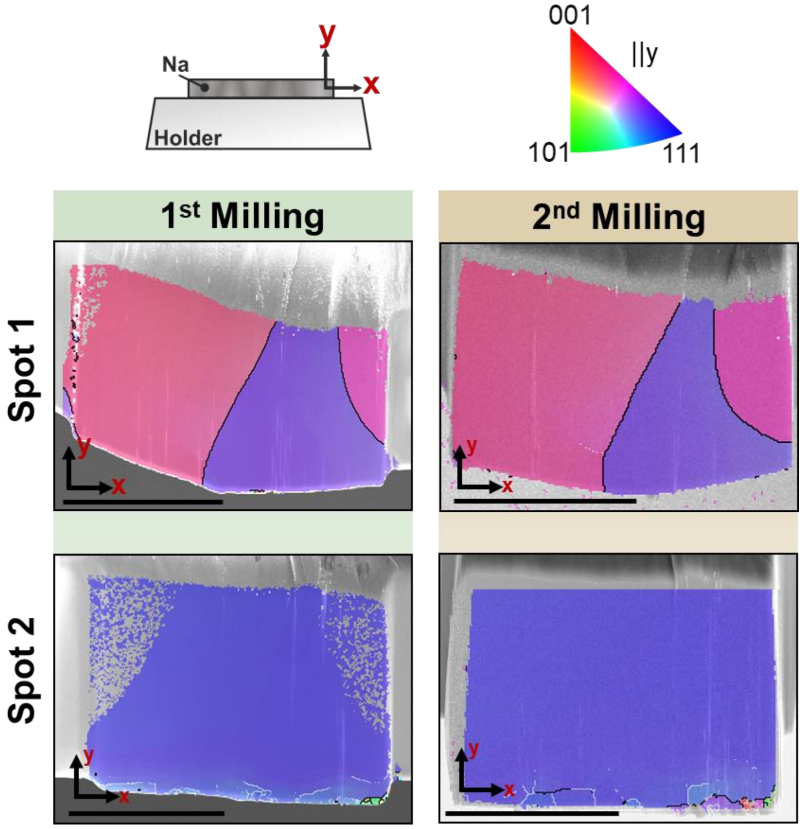

Figure S7. *Inverse pole figure of Q-Na foil after preparation of a cross-section via focused-ion beam and a second milling at two different spots. The IPF are given parallel to the y-direction (milling direction) as indicated in the schemes. Black lines indicate large angle grain boundaries (>10°) while misorientations between 2° and 10° are indicated by white lines. The scale bar equals 200 µm in each image. At both spots, no changes in grain orientation and grain size are observed after a second milling step, demonstrating that the FIB preparation does not affect the microstructure of sodium.*

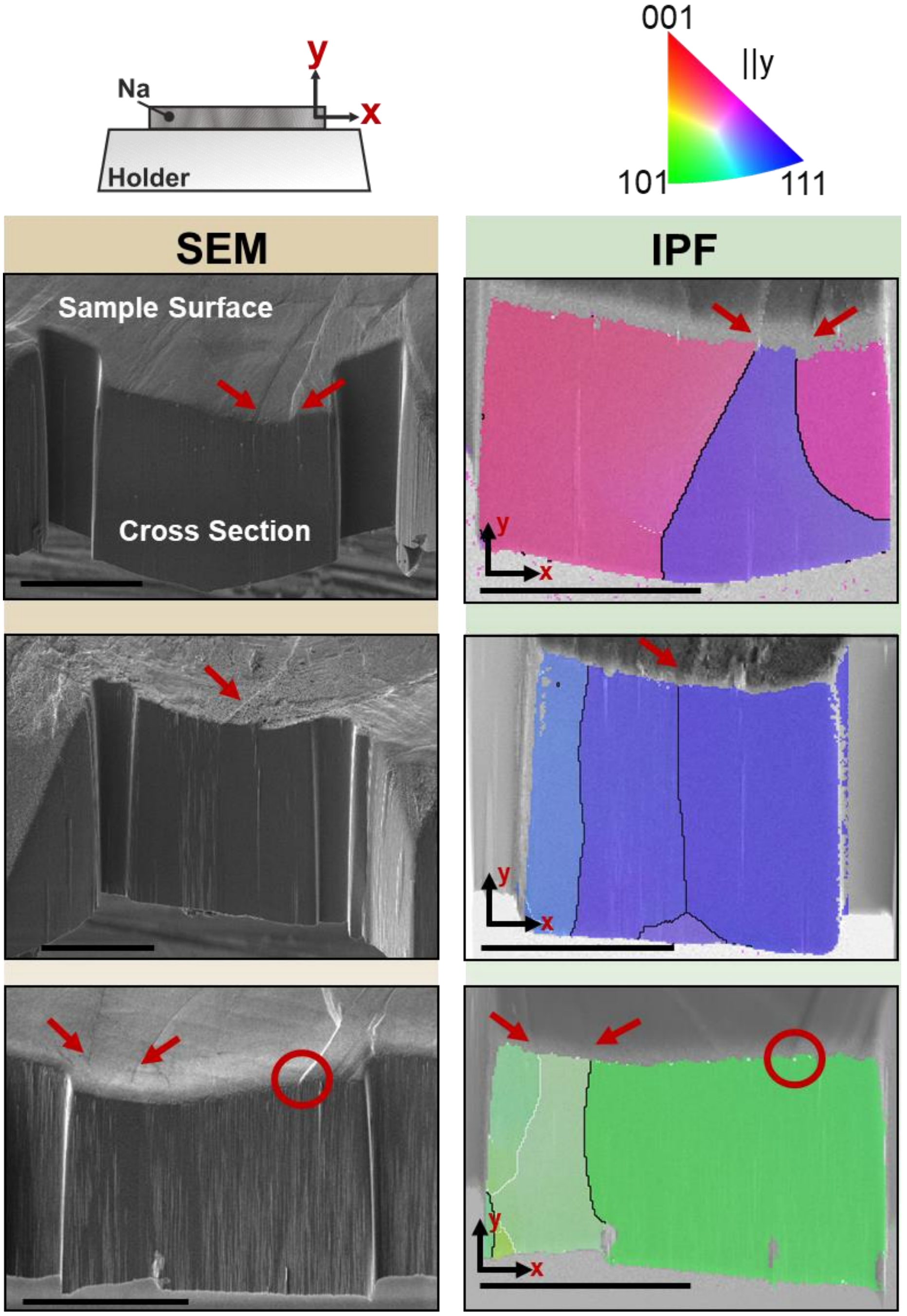

Figure S8. *SEM images of three different cross-sections of sodium foils prepared via focused-ion beam milling (left column) and the corresponding IPF (right column). IPFs are given parallel to the y-direction (milling direction) as illustrated by the schemes in the top of the figure. Large angle boundaries (>10°) are indicated by black lines while misorientations between 2° and 10° are indicated by white lines. In the SEM images (left column), similar lines (highlighted by red arrows) can be seen on the sample surface as already described in Figure 1 (main text). A cross-section perpendicular to the line profile with subsequent microstructure analysis (IPF in the right-hand column) shows grain boundaries along the observed lines in the volume of the sodium film. The identified line on the sodium surface correlates with the presence of a grain boundary. It should be noted that a line on the surface does not necessarily indicate the presence of a grain boundary, as shown in the example circled in red. The evaluation of the line structure on a foil surface is insufficient for an accurate mapping and determination of the grain size.*

4 Impedance Spectra of Electrodeposited Alkali Metals at CC|SE Interface

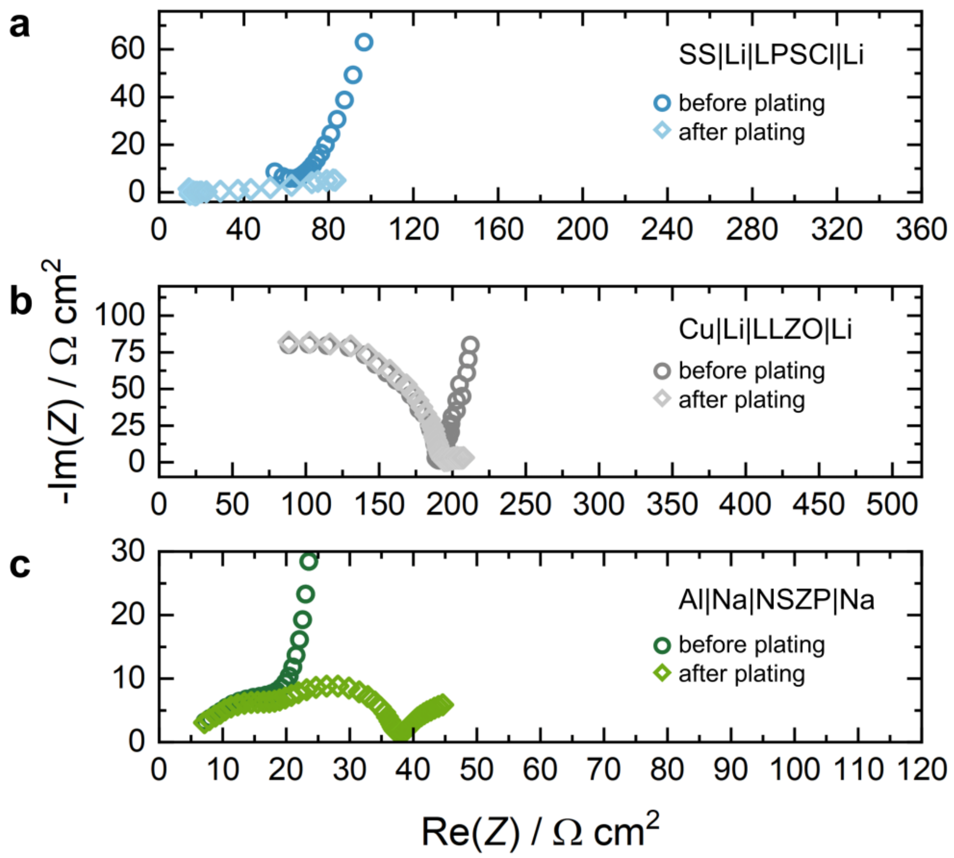

Figure S9. *Impedance spectra before and after electrodeposition of lithium at the SS|LPSCl and Cu|LLZO interface as well as sodium at the Al|NZSP interface. In all cases, a characteristic shift from a blocking electrode impedance to a typical metal electrode impedance can be observed. In the case of SS|LPSCl, a short-circuit occurred, which explains the low resistance and uncommon shape of the impedance spectrum.*

5 Cross-sectional Inverse Pole Figures of Electrodeposited Alkali Metal

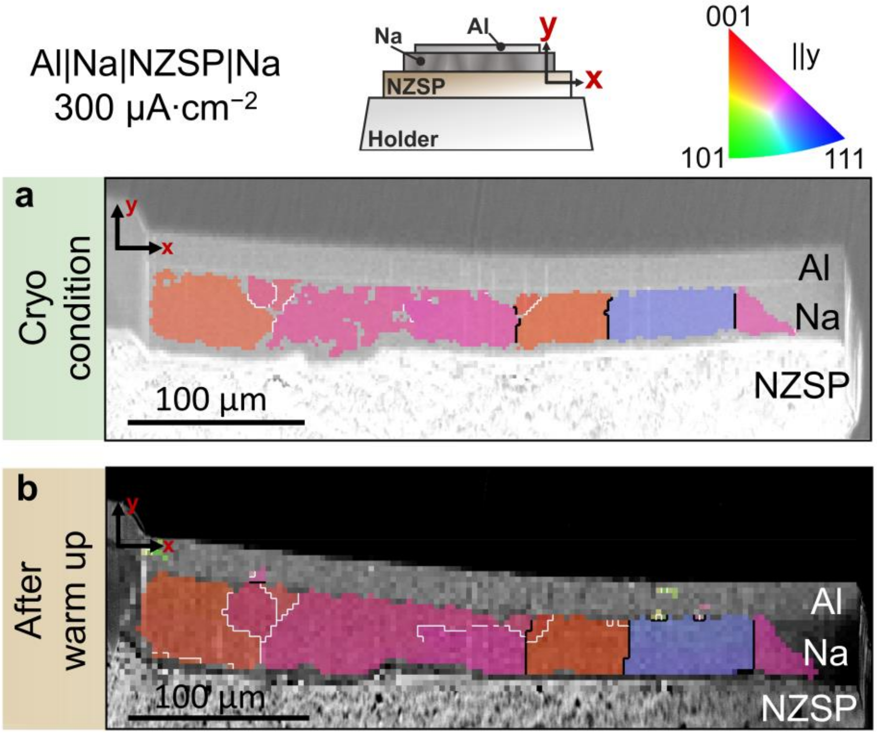

Figure S10. *a) Inverse pole figure of electrodeposited sodium (300 µA cm^{-2}) at an interface between a carbon-coated aluminum current collector and NZSP solid electrolyte. The microstructure of the deposited sodium was characterized under cryogenic conditions. b) Inverse pole figure of sodium after warm up of the specimen to room temperature. The IPF of NZSP and aluminum are not shown. The IPF of sodium is presented parallel to the y-direction (growth direction) as visualized by the schemes. Black lines represent large angle grain boundaries (>10°) while misorientations between 2° and 10° are indicated by white lines. The deposited sodium consists of grains on the order of tens of microns. In addition, large grain boundaries are preferentially observed parallel to the deposition direction, i.e., perpendicular to the surface of the NZSP. No changes in grain size or grain boundary orientation were observed after warming the specimen, indicating that no detrimental grain growth by annealing occurred.*

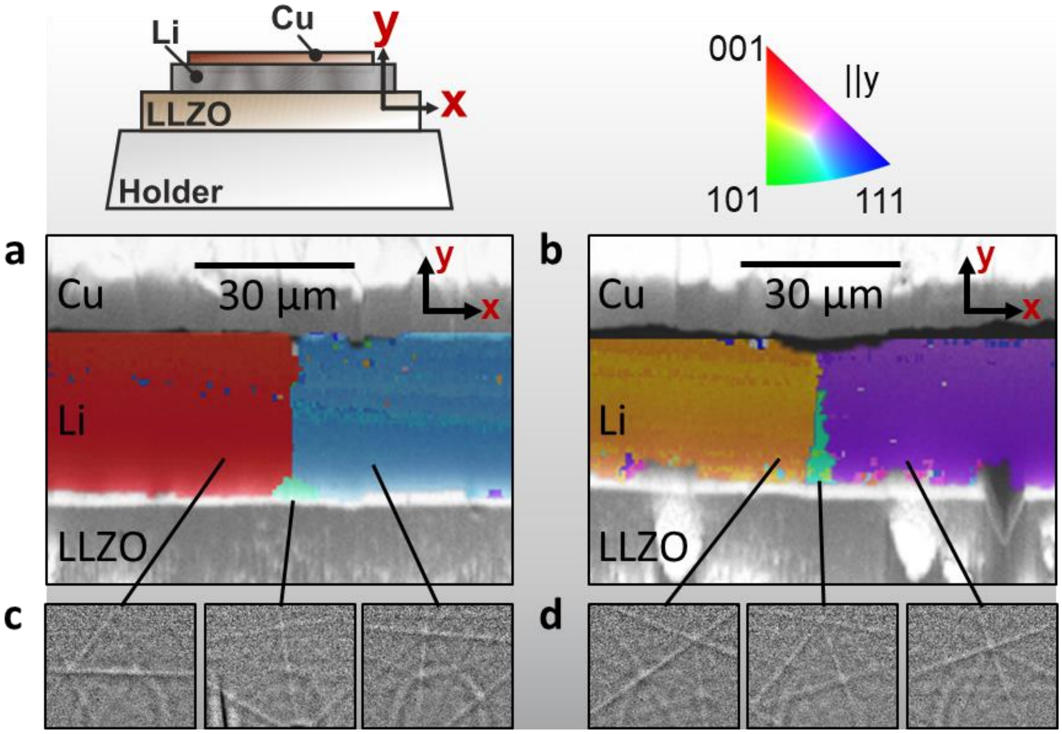

Figure S11. *Magnified IPF maps of different areas of the Cu|Li|LLZO sample shown in Figure 3c are depicted in a) and b). A selected EBSP for each grain is shown in c) and d) confirming the different grain orientations. The small green grains likely exhibit a smaller rate of growth after emergence, which is why the neighboring, faster growing grains constrict the space for the green grains for further growth at higher film thicknesses.*

6 *Influence of SE or CC Microstructure on Electrodeposited Metal Microstructure*

It is not clear what sample property or deposition parameter primarily governs the deposited metal microstructure, such as grain size and microstructure of SE and CC. To check for a relationship between the substrate microstructure with the deposited microstructure, IPF maps of lithium plated on a copper CC with both layers being indexed are shown in **Figure S12a**. Although electrodeposited lithium preferentially deposits on the Cu(111) crystal facet in liquid electrolyte, we found no clear relation between the grain size and orientation of the copper and lithium at the Cu|SE interface.[1] This uncorrelated growth may be due to surface passivation or solid electrolyte interphase (SEI) formation masking the underlying grain structure. A partial masking of preferred deposition sites due to passivation was also reported for lithium growth on copper substrates when different liquid electrolyte with different reactivity were used.[2] In general, it is unlikely for lithium to undergo epitaxial growth on copper due to a mismatch-induced lattice strain (a_{Cu} = 3.6 Å vs. a_{Li} = 3.5 Å).[3,4] The minor effect of the CC microstructure on the resulting grain structure becomes even clearer when lithium is electrodeposited on a Q-Li reservoir foil, as shown by the IPF map in **Figure S12b**. In this case, neither the grain size nor the grain orientation of the electrodeposited grains matches that of the Q-Li reservoir. Furthermore, the size of the solid electrolyte grains (see **Figure S13**) does not appear to have a direct correlation with the microstructure of either electrodeposited alkali metal, as the size difference is quite large. It is therefore likely that the deposit microstructure is dominated by the nucleation process and grain growth itself.

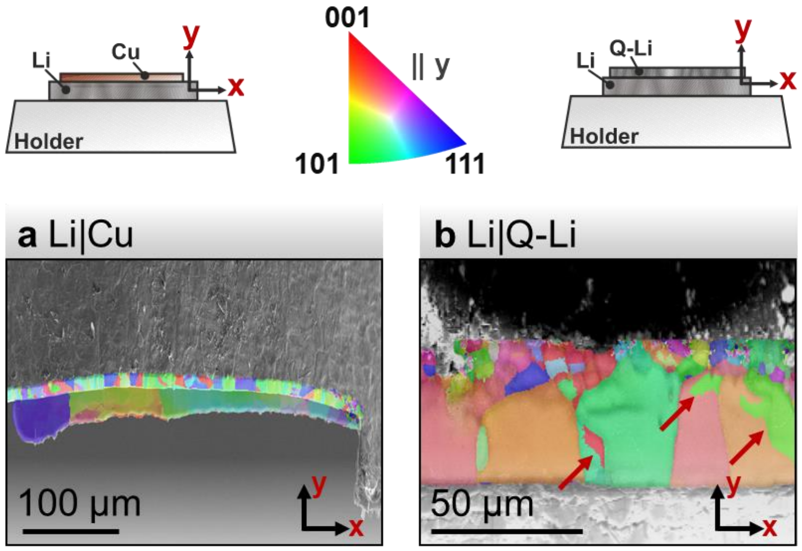

Figure S12. *Alkali metal plated on different current collectors. IPF maps of lithium plated on a copper CC or a Q-Li reservoir are depicted in a) and b), respectively. No correlation between the*

microstructure of the CC or reservoir to the electrodeposited metal is found in any case. Red arrows indicate areas where grains are partly indexed incorrectly due to crystallographic pseudosymmetry.

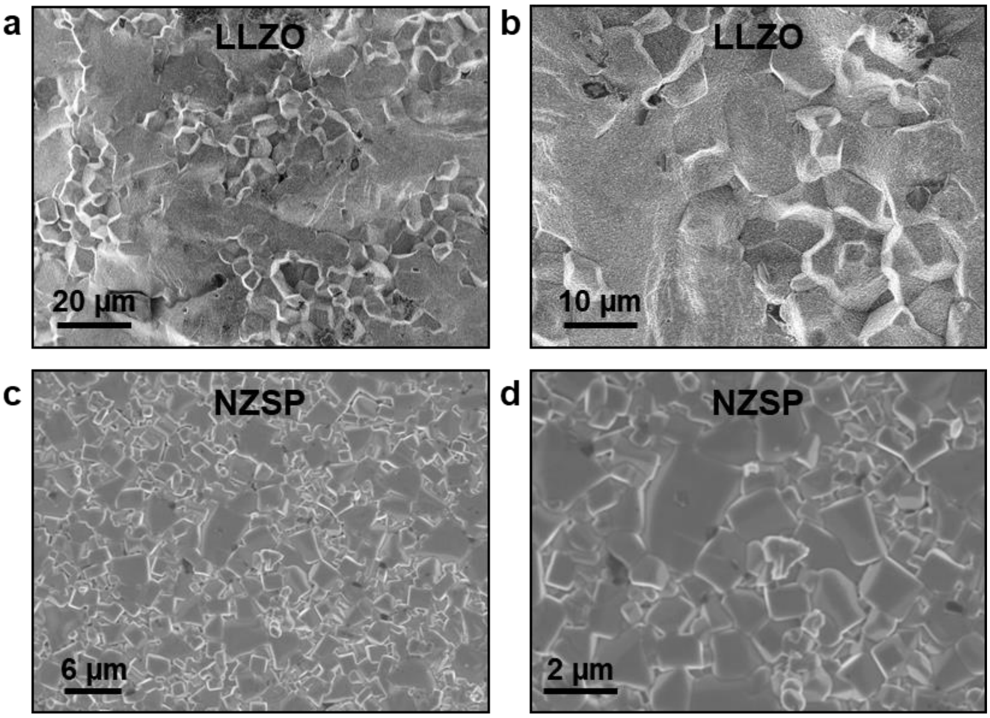

Figure S12. *Scanning electron microscopy images of the surface of LLZO and NZSP. The surface of LLZO is shown in a) and magnified in b) showing a grain size of roughly 5 µm to 20 µm. The grain size of NZSP is in the range of 0.5 µm to 4 µm, as depicted in c) und d).*

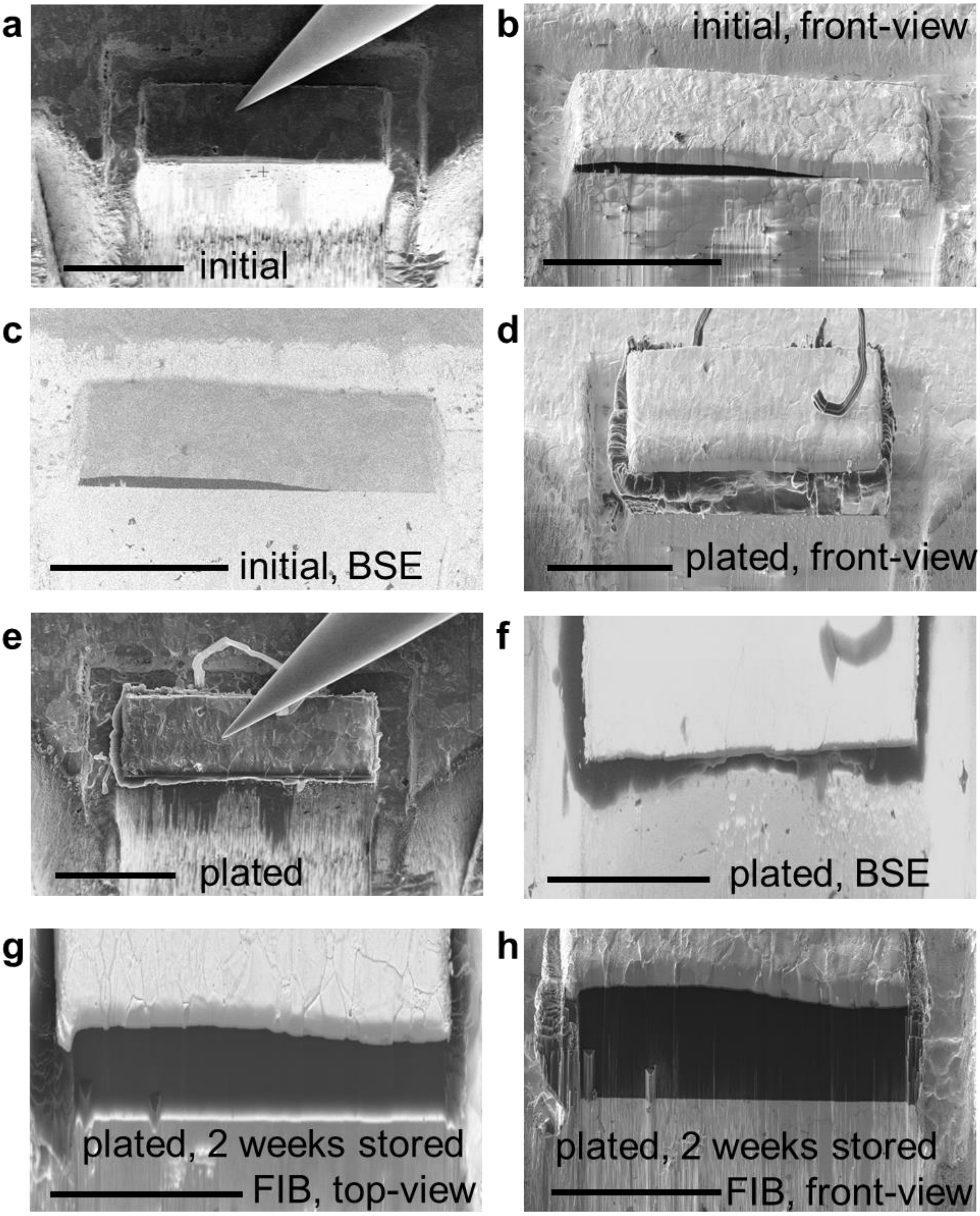

***Figure S14**. Overview SEM images prior and after lithium plating during the in situ EBSD experiment are shown in a) and b). Front-view images after tilting the sample to 55° (including the tilted sample holder) are shown in c) and d). A lithium thickness of around 40-50 µm was achieved dependent on the measurement spot, which fits well to the passed charge. The apparent different aspect ratio of the electrode patch is explained by an uneven film-growth, with higher thicknesses to the back of the patch. BSE images to prove the existence of low-density lithium are shown in e) and f) accordingly. Further, g) and h) show SEM images after two weeks of storage and renewed interface polishing via FIB. All scale bars are equal to 100 µm.*

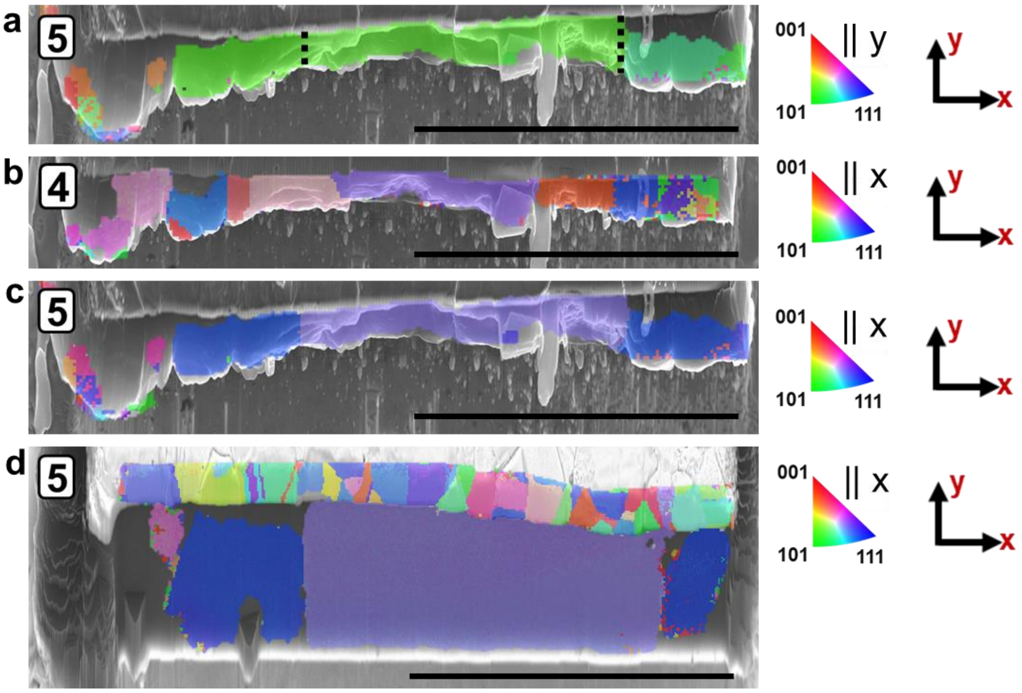

Figure S15. *Additional IPF maps to* ***Figure 4c****. a) shows the IPF map in y-direction directly after plating but without additional FIB polishing, confirming the unchanged microstructure during the two-week storage period afterward. Further, b) and c) show the maps of step 4 and 5 also in x-direction to get a better overview of present grain orientations. The map provided in Figure 4c (5) is further shown in x-direction in d). After polishing, the copper grains are also indexed, but do not show a significant influence on the lithium microstructure. All scale bars equal 100 µm.*

8 In situ Sodium Electrodeposition at the Q-Na|NASICON Interface

An overview of the electrodeposition of sodium on a pre-existing sodium reservoir is depicted in **Figure S16**. In contrast to lithium, the experiment was conducted using a Q-Na|NZSP|Na cell with an initial electrode thickness of ~50 µm. A smooth and flat voltage plateau is observed during deposition of roughly 1 mAh cm^{-2}. Assuming uniform deposition across the electrode, the deposited capacity corresponds to a sodium layer thickness of approximately 9 µm, which fits to the observed thickness increase at the cross-section shown in **Figure S16b-d**. Prior to deposition, three main grains are observed at the cross-section, while a few small grains are located directly at the interface (**Figure S16e**). Similar to the observation for lithium in **Figure 4c**, the large grain (green) with an orientation close to <101> grows vertically during deposition and consumes the neighboring grains with orientations close to <111>, which results in lateral grain boundary motion. Moreover, the thickness of the smaller grains on the left side of the cross-section directly at the interfaces slightly increases during deposition. The identification of newly formed grains is challenging, as the sodium partly grows out of the image plane leading to shadowing of the interface. However, a new grain appears on the right side of the cross-section as shown by a red arrow in **Figure S16g**. After finishing the deposition experiment, the cross-section was polished via FIB uncovering a second formed grain indicated by red arrows in **Figures S16f** and **S16h**. It should be noted that after polishing, the new cross-section is approximately shifted 10 µm compared to the previous plane, making it difficult to establish an unambiguous correlation. However, upon checking the EBSPs for these grains in **Figures S16i** and **Figure S16j**, it can be concluded that this is the same grain. Similar to the deposition of lithium, the grain width of sodium changes during deposition and grains in the <101> orientation show preferential growth. Although the microstructure of the discussed cross-section has not been characterized after a defined storage time, it is unlikely that the grain width has changed during storage, as shown by the example in **Figure S10**. Thus, the microstructural evolution of sodium likely follows that of lithium.

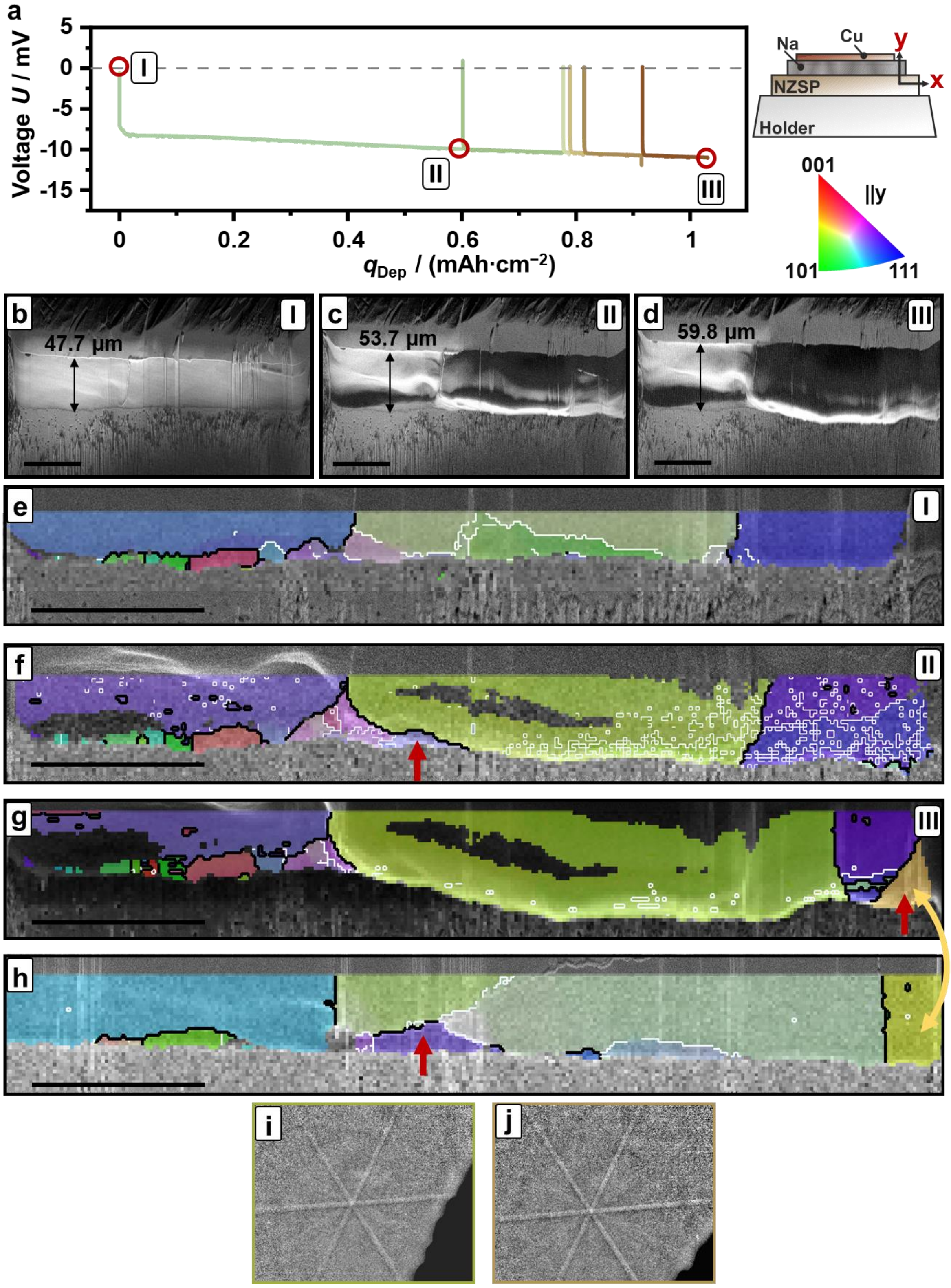

Figure S16. *In situ EBSD of electrodeposited sodium at a Q-Na/NZSP interface. The deposition was performed inside the SEM using a current density of 300 µA cm^{-2}. The voltage profile of the deposition is shown in a). The voltage spikes indicate the start of each deposition step. EBSD characterization is indicated by a red circle. Forward Scatter Electron images (FSE) of the pristine cross-section and after the two deposition steps are shown in b-d. During each deposition step a layer increase of roughly 5 µm – 6 µm is observed. The corresponding IPF maps of the interface are visualized in e-g. IPF maps are given parallel to the growth direction. Black lines indicate large angle grain boundaries (>10°). White lines visualize misorientations between 2° and 10° of neighbor*

pixels. After the finishing deposition the cross-section was polished via FIB and again characterized by EBSD. The corresponding IPF map is shown in h). Red arrows indicate newly formed grains by electrodeposition. Despite different color in the IPF maps, the highlighted grains by the yellow arrow are similar, as indicated by the pattern in i) and j). The different color originates from pseudosymmetry caused by dynamic pattern simulation. The scale bar indicates 50 µm.

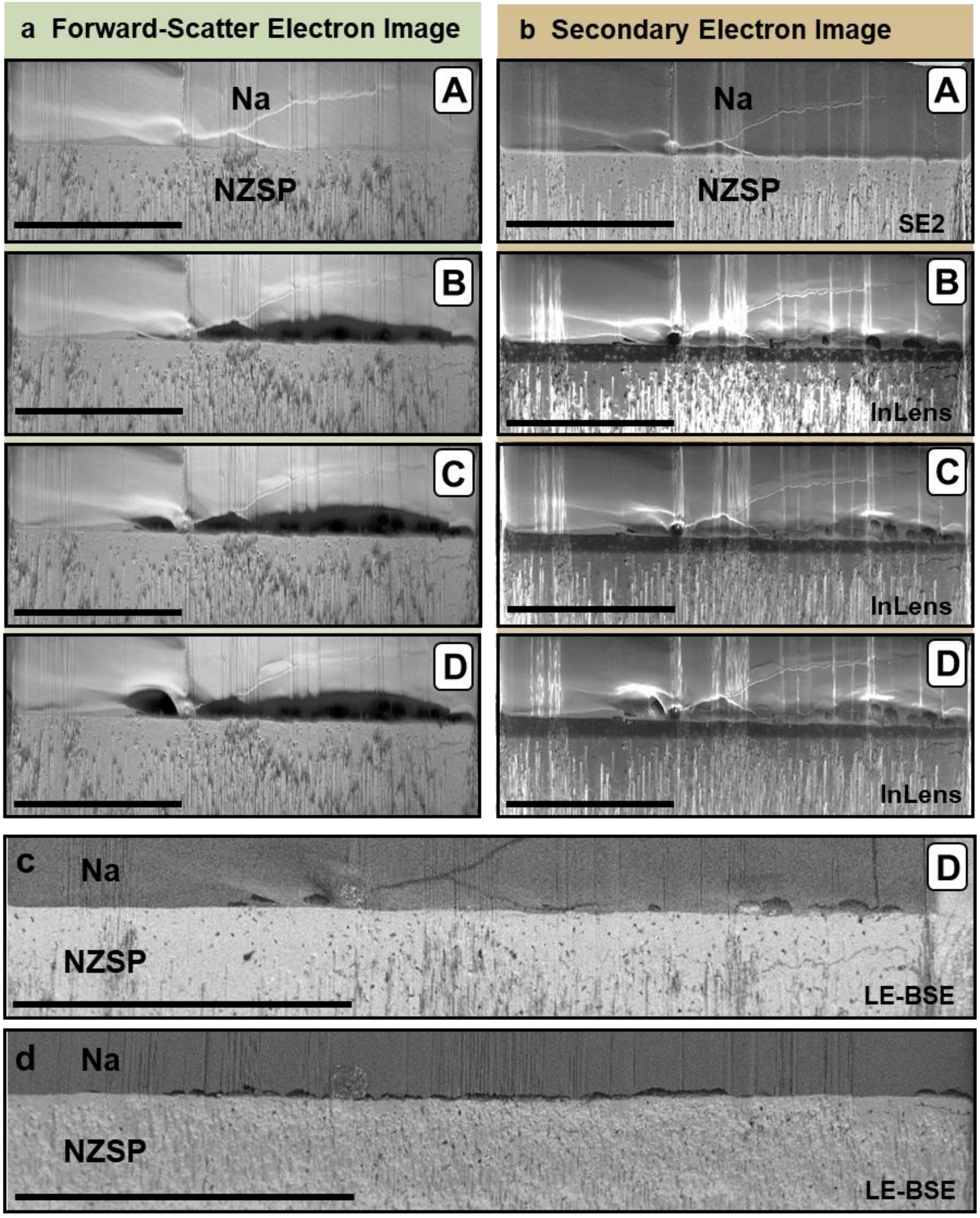

***Figure S17**. Compilation of electron images during stripping of sodium at Na/NSZP interface. The given notation (A) to (D) corresponds to the given voltage profile in **Figure 4b**. During the in situ experiment the cross-section was imaged at an angle of 70° using a) forward scatter electrons and b) secondary electrons. After the stripping experiment the cross-section was imaged using a low energy-backscatter electron detector (LE-BSE) in an angle of 35°. Based on the different geometries the pore shape and size differ as shown in c). Afterwards the cross-section was again polished revealing unambiguous pores at the Na/NSZP interface. As polishing shifts the cross-section plane roughly 10 µm behind the previous one, no perfect correlation of pores between c) and d) is observed. Scale bars equal 100 µm*

10 References

1. Ishikawa, K., Ito, Y., Harada, S., Tagawa, M. & Ujihara, T. Crystal Orientation Dependence of Precipitate Structure of Electrodeposited Li Metal on Cu Current Collectors. *Cryst. Growth Des.* **17**, 2379–2385 (2017).

2. Kim, Y. J. *et al.* Facet selectivity of Cu current collector for Li electrodeposition. *Energy Storage Mater.* **19**, 154–162 (2019).

3. Nadler, M. R. & Kempter, C. P. Crystallograpic data 186. lithium. *Crystallogr. Data* **31**, 2109 (1959).

4. Davey, W. P. Precision Measurements of the Lattice Constants of Twelve Common Metals. *Phys. Rev.* **25**, 753–761 (1925).
